# Dendrimer-doxorubicin conjugates exhibit improved anticancer activity and reduce doxorubicin-induced cardiotoxicity in a murine hepatocellular carcinoma model

**DOI:** 10.1371/journal.pone.0181944

**Published:** 2017-08-22

**Authors:** Sibu P. Kuruvilla, Gopinath Tiruchinapally, A. Colleen Crouch, Mohamed E. H. ElSayed, Joan M. Greve

**Affiliations:** 1 Department of Materials Science and Engineering, University of Michigan, Ann Arbor, MI United States of America; 2 Department of Biomedical Engineering, University of Michigan, Ann Arbor, MI United States of America; 3 Department of Mechanical Engineering, University of Michigan, Ann Arbor, MI United States of America; 4 Program of Macromolecular Science and Engineering, University of Michigan, Ann Arbor, MI, United States of America; University of Catanzaro, ITALY

## Abstract

Hepatocellular carcinoma (HCC) is the 2^nd^ leading cause of cancer-related deaths every year globally. The most common form of treatment, hepatic arterial infusion (HAI), involves the direct injection of doxorubicin (DOX) into the hepatic artery. It is plagued with limited therapeutic efficacy and the occurrence of severe toxicities (e.g. cardiotoxicity). We aim to improve the therapeutic index of DOX delivered via HAI by loading the drug onto generation 5 (G5) poly(amidoamine) (PAMAM) dendrimers targeted to hepatic cancer cells via N-acetylgalactosamine (NAcGal) ligands. DOX is attached to the surface of G5 molecules via two different enzyme-sensitive linkages, L3 or L4, to achieve controllable drug release inside hepatic cancer cells. We previously reported on P1 and P2 particles that resulted from the combination of NAcGal-targeting with L3- or L4-DOX linkages, respectively, and showed controllable DOX release and toxicity towards hepatic cancer cells comparable to free DOX. In this study, we demonstrate that while the intratumoral delivery of free DOX (1 mg/kg) into HCC-bearing nod scid gamma (NSG) mice achieves a 2.5-fold inhibition of tumor growth compared to the saline group over 30 days, P1 and P2 particles delivered at the same DOX dosage achieve a 5.1- and 4.4-fold inhibition, respectively. Incubation of the particles with human induced pluripotent stem cell derived cardiomyocytes (hiPSC CMs) showed no effect on monolayer viability, apoptosis induction, or CM electrophysiology, contrary to the effect of free DOX. Moreover, magnetic resonance imaging revealed that P1- and P2-treated mice maintained cardiac function after intraperitoneal administration of DOX at 1 mg/kg for 21 days, unlike the free DOX group at an equivalent dosage, confirming that P1/P2 can avoid DOX-induced cardiotoxicity. Taken together, these results highlight the ability of P1/P2 particles to improve the therapeutic index of DOX and offer a replacement therapy for clinical HCC treatment.

## Introduction

**Fig 1:**
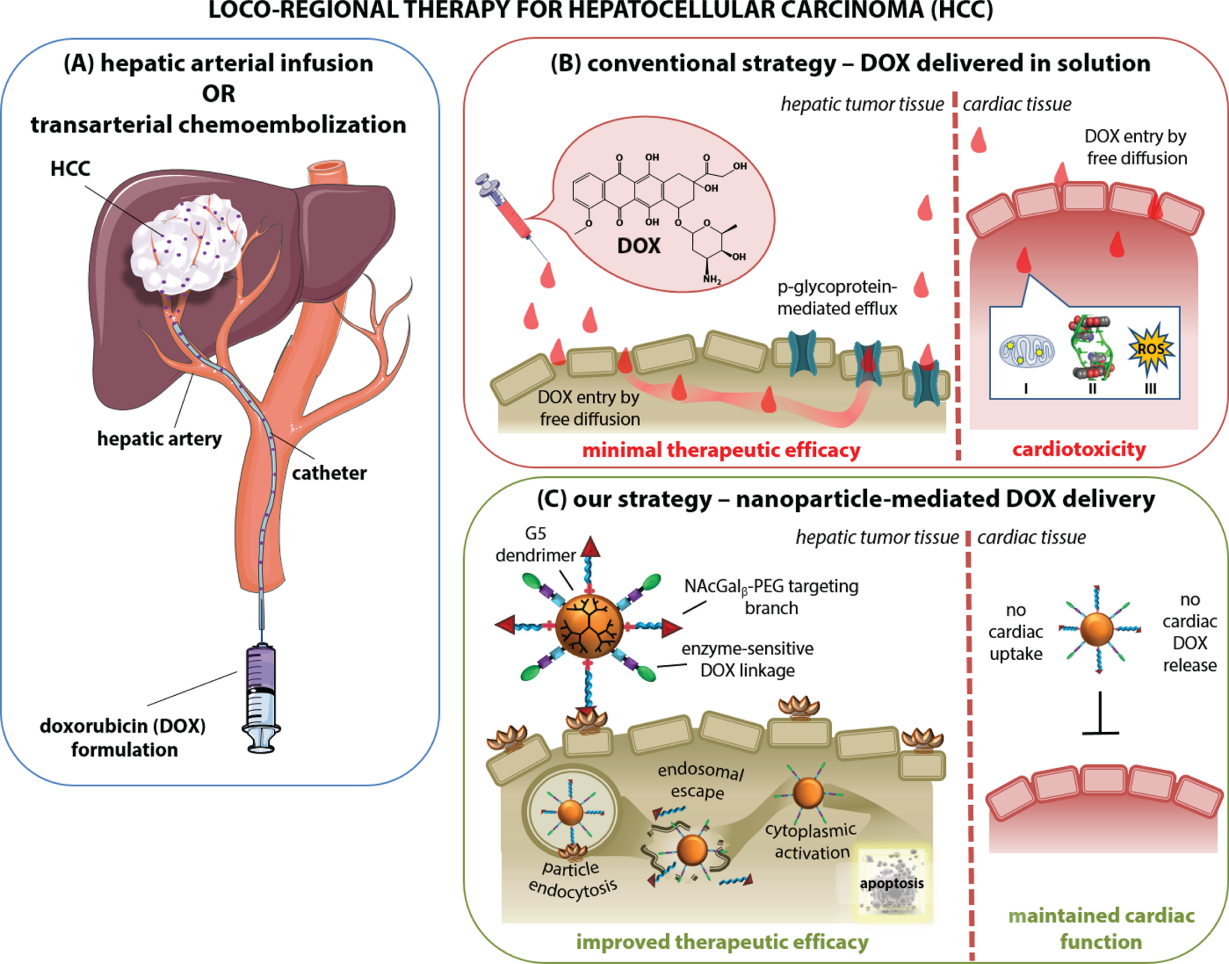
Strategy for improving loco-regional therapy for hepatocellular carcinoma. (**A**) Conventional therapies for hepatocellular carcinoma (HCC), such as hepatic arterial infusion (HAI) or trans-arterial chemoembolization (TACE), involve the direct injection of doxorubicin (DOX) into the hepatic artery. (**B**) The delivery of free doxorubicin in solution through HAI/TACE is limited by minimal therapeutic response, due to p-glycoprotein-mediated efflux of the drug, and severe cardiotoxicity as a result of **I**) mitochondrial iron accumulation, **II**) topoisomerase II inhibition, and **III**) generation of reactive oxygen species (ROS). (**C**) Our strategy involves the delivery of DOX via enzyme-activated nanoparticles targeted to hepatic cancer cells to achieve antitumor activity while avoiding DOX-induced cardiotoxicity. Specifically, NAcGal ligands displayed on the particle target hepatic cancer cells via the asialoglycoprotein receptor (ASGPR) and are internalized by receptor-mediated endocytosis. The particles are then able to escape the endosome into the cytoplasm where hepatic azoreductase enzymes cleave DOX from the particle, allowing it to induce its cytotoxic behavior through mechanisms **I**, **II**, and **III**. The combination of receptor-mediated targeting and hepatic enzyme-mediated DOX release prevent distribution and toxicity from occurring in the heart.

Worldwide, hepatocellular carcinoma (HCC) is the 5^th^ most commonly-occurring cancer and the 2^nd^ leading cause of cancer-related deaths every year. Currently, due to the low percentage of HCC patients that are eligible for surgery (9–29%)[1] and the high rates of tumor recurrence after resection (60%),[2] loco-regional chemotherapy delivered through hepatic arterial infusion (HAI) or transarterial chemoembolization (TACE) is the first-line treatment for advanced HCC. HAI involves the direct injection of a chemotherapeutic drug (e.g. doxorubicin; DOX) into the hepatic artery (**Fig 1**), given that the hepatic artery is the primary tumor-feeding vessel in clinical HCC. TACE is a modification of HAI in which DOX is delivered as a suspension in an embolizing agent (e.g. lipiodol) to simultaneously induce ischemia in the tumor. Unfortunately, however, HAI procedures have experienced limited antitumor activity in several clinical trials delivering DOX by itself or as part of a cocktail (e.g. drug-eluting microbeads).[3,4] In fact, HAI response rates do not exceed 15% for the most advanced HCC stages (Child-Pugh score B/C),[5,6] and the survival advantage over supportive care alone has been small (3.1 to 4.8 weeks).[3,7]

One primary driver of the limited therapeutic response of DOX delivered by HAI is the expression of p-glycoprotein (P-gp) in hepatocellular carcinoma.[8,9] The reliance on free diffusion to achieve high intracellular concentrations of DOX creates a large gradient within the cell of DOX, with the highest concentration being at or near the cell surface where P-gp is located. The basal expression of P-gp facilitates efflux of DOX out of the cell, minimizing its intracellular concentration (**Fig 1**).[10] Further, repeated DOX exposure leads to the upregulation of P-gp, conferring resistance to the cell that leads to the high rates of tumor recurrence observed clinically once chemotherapy is removed.[2,11,12]

Despite the local administration of DOX through HAI, P-gp efflux combined with the need to deliver high doses of DOX leads to high systemic concentrations of the drug. This systemic concentration leads to off-target toxicities, namely cardiotoxicity,[7,13,14] hepatic dysfunction,[4,15] myelosuppression,[4] and portal vein thrombosis.[16] The severity of toxicities developing from DOX administration limits the dosage that can be used, further incapacitating the efficacy of DOX by lowering its cumulative dose against the tumor.[2] The primary reason for high rates of hepatic damage is the reliance on free diffusion for DOX penetration into the cell, leading to non-specific internalization and significant concentrations of DOX in healthy hepatic tissue that compromise the reserved liver function.[17,18] Complementarily, ischemia of healthy hepatic tissue induced by lipiodol used in TACE further advances the hepatic damage.[19] It is therefore not surprising that HAI/TACE procedures are not recommended for patients with Child-Pugh classification late B or C due to their already-compromised liver function and the intolerable hepatic dysfunction that would occur with DOX therapy.[4,6,15]

The most severe clinical side effect of DOX administration, however, is cardiotoxicity. Release of DOX into the systemic circulation after injection by HAI enables DOX accumulation in the heart by free diffusion (**Fig 1**).[17,18] Cardiomyocytes are a primary target of DOX-induced oxidative stress due to their high reliance on oxidative substrate metabolism, which makes them vulnerable to mitochondrial DOX-iron complexing and subsequently, reactive oxygen species (ROS) generation.[13] On top of ROS, inhibition of topoisomerase II and mitochondrial iron accumulation contribute to cardiomyocyte damage, dysfunction, and apoptosis.[13,14,20] The development of cardiotoxicity in patients receiving DOX ranges from 4–100% depending greatly on the cumulative dose administered,[7,13,21,22] but the patients that are affected face mortality rates between 50–100%.[21–23] There is an urgent need to develop a drug delivery system that can specifically deliver DOX to hepatic cancer tissue to achieve comparable efficacy to the free drug, but avoid the systemic release that results in significant side effects like cardiotoxicity.

**Fig 2:**
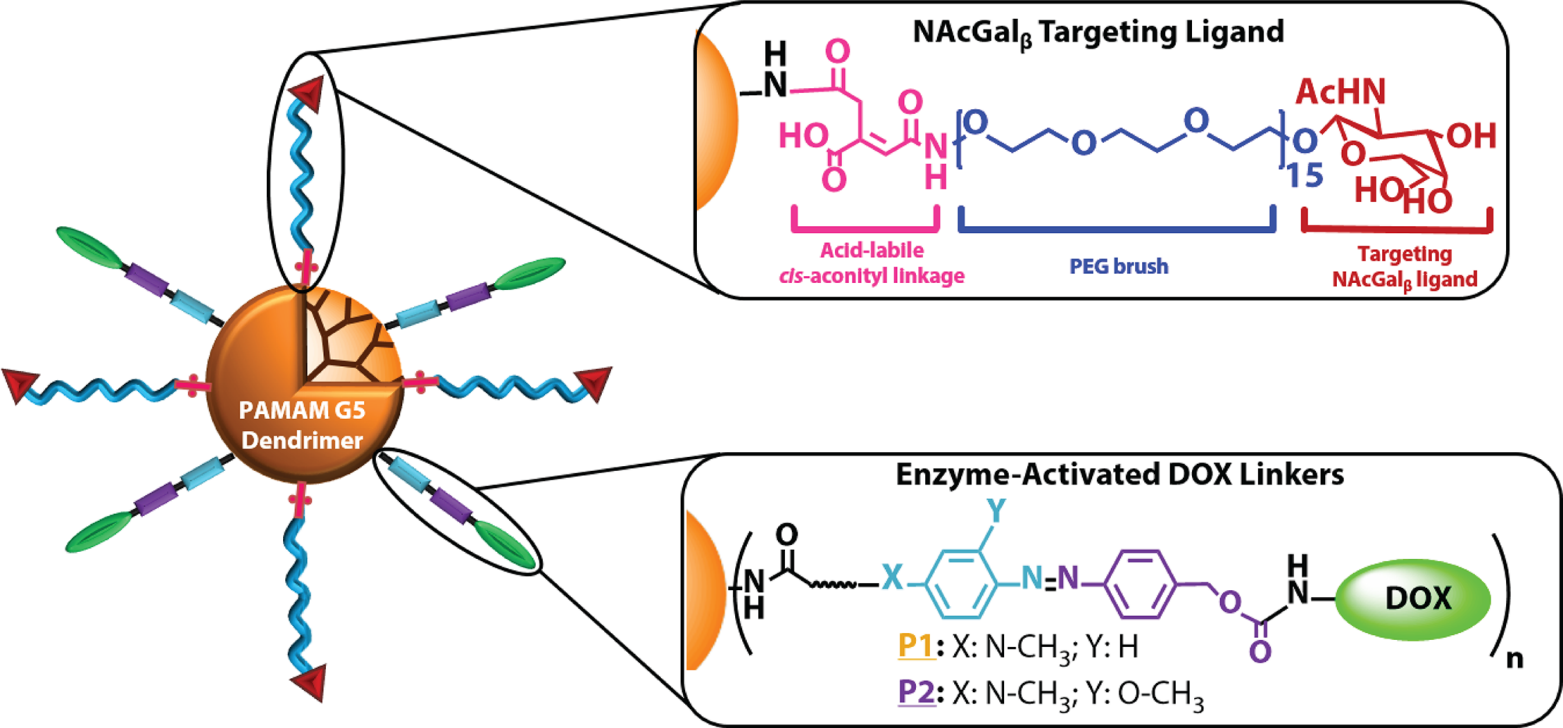
Schematic of P1 and P2 particles. G5 PAMAM dendrimers are functionalized with N-acetylgalactosamine (NAcGal)_β_-terminated PEG brushes attached to G5 via an acid-labile cis-aconitic (*c*) spacer to facilitate selective binding to the asialoglycoprotein receptor (ASGPR) overexpressed on hepatic cancer cells. Doxorubicin (DOX) molecules are also attached via two different enzyme-sensitive linkages to form either **P1** [(NAcGal_β_-PEG*c*)_16.6_-G5-(L3-DOX)_11.6_] or **P2** [(NAcGal_β_-PEG*c*)_16.6_-G5-(L4-DOX)_13.4_] particles.

We reported the development of enzyme-activated polymer-DOX nano-conjugates as a potential new therapy for HCC (**Fig 1**).[24–27] Specifically, we conjugated DOX molecules to water-soluble generation 5 (G5) of poly(amidoamine) (PAMAM) dendrimers via aromatic azo-linkages forming G5-DOX conjugates, which are selectively recognized and cleaved by azoreductase enzymes expressed by hepatic cancer cells (**Fig 2**).[26] Targeting hepatic cancer cells was achieved by conjugating *N*-acetylgalactosamine (NAcGal_β_) sugar molecules to the free tip of a PEG brush anchored to the G5 surface via acid-labile cis-aconityl (*c*) linkages (**Fig 2**), which proved to achieve rapid internalization into hepatic cancer cells while avoiding recognition and uptake by healthy hepatocytes.[25,27] In these reports, we describe the synthesis of two NAcGal_β_-targeted nano-conjugates where DOX molecules are attached to the G5 surface via two different enzyme-sensitive azo-linkages (i.e. **L3**-DOX or **L4**-DOX) yielding NAcGal_β_-PEG*c*-G5-**L3**-DOX or NAcGal_β_-PEG-G5-**L4**-DOX, named P1 and P2, respectively (**Fig 2**). We hypothesize that P1 and P2 particles are able to avoid P-gp-mediated efflux of DOX due to receptor-mediated delivery instead of passive diffusion, which has been shown to improve intracellular concentrations of DOX within cancer cells and increase antitumor activity.[10] Further, the specific targeting combined with enzyme-mediated DOX release should mitigate the occurrence of systemic toxicities such as cardiac toxicity. In this manuscript, we report the antitumor activity of P1 and P2 particles after direct intratumoral (i.t.) injection into nod scid gamma (NSG) mice bearing ectopic HepG2 tumors. We also investigated the cardiac toxicity observed with P1 and P2 particles compared to equal doses of free DOX in plated cardiomyocytes or after intraperitoneal (i.p.) administration in NSG mice. Quantifying the antitumor activity as well as the induced cardiotoxicity of P1 and P2 particles *in vivo* will elucidate their potential as a translational DOX formulation for HCC therapy.

## Materials and methods

### Materials

G5-(NH_2_)_128_ dendrimers with a diaminobutane core were purchased from Andrews ChemServices (Berrien Springs, MI) and purified by dialysis against deionized water using Slide-A-Lyzer dialysis cassettes (MWCO 10 kDa, Thermo Fisher Scientific, Rockford, IL) to remove imperfect dendrimers and debris. Doxorubicin-HCl was purchased from AvaChem Scientific (San Antonio, TX). *N*-acetylgalactosamine, 4-pentynoic acid, pyridine, trimethylphosphine solution (1.0 M in THF), triethylamine (TEA), acetic anhydride (Ac_2_O), 1-ethyl-3-(3-dimethylaminopropyl) carbodiimide hydrochloric acid (EDC.HCl), benzotriazol-1-ol (HOBt), trifluoroacetic acid (TFA), bathophenonthroline sulfonated sodium salt (SBP), copper bromide (CuBr), anhydrous dimethylsulfoxide (DMSO), anhydrous dichloromethane (DCM), anhydrous dimethylformamide (DMF), anhydrous tetrahydrofuran (THF), *cis*-aconitic anhydride (cis-Ac), and bovine serum albumin (BSA) were purchased from Sigma-Aldrich Inc. (St. Louis, MO). Trimethylsilyl trifluoromethanesulfonate (TMSOTf), N,N-diisopropyl ethyl amine (DIPEA), camphor sulphonic acid (CSA), sodium azide (NaN_3_), sodium ascorbate, and benzotriazol-1-yl-oxytripyrrolidinophosphonium hexafluorophosphate (PyBOP) were purchased from Across Organics Chemicals (Geel, Belgium). N-hydroxysuccinimide-poly(ethylene glycol)-Boc (2 kDa) was purchased from JenKem Technology USA Inc (Plano, TX). 2-{2-(2-Chloroethoxy)ethoxy}ethanol was purchased from TCI America (Portland, OR). Dialysis cassettes (MWCO 1–10 kDa) were purchased from Thermo Fisher Scientific (Rockford, IL). Minimum essential medium (MEM), OPTI-MEM reduced serum medium, fetal bovine serum (FBS), 0.25% trypsin/0.20% ethylenediaminetetraacetic acid (EDTA) solution, phosphate buffered saline (PBS), penicillin/streptomycin/amphotericin solution, sodium pyruvate, minimum non-essential amino acid (NEAA) solution, and 0.4% trypan blue solutions were purchased from Life Technologies (Thermo Fisher Scientific, Rockford, IL).

### Synthesis and characterization of P1 and P2 particles

We previously reported the synthetic techniques used to create P1 and P2 particles.[27] We used similar techniques to fabricate the particles presented here, and the complete synthesis along with nuclear magnetic resonance (NMR) and time-of-flight matrix-assisted laser desorption/ionization (MALDI) spectra can be found in the **Supporting Information**. We relied on NMR and MALDI data to establish the molecular weights of P1 and P2 particles, as well as the density of loaded NAcGal_β_-PEG*c* and L(x)-DOX linkages.

We measured the particle size of P1 and P2 particles by dynamic light scattering (DLS) using a 90Plus particle size analyzer (Brookhaven Instruments, Holtsville, NY). The particles were diluted in DI water at 1:20 v/v with 10% tween 20 in order to limit nanoparticle aggregate formation. After sonication for 20 minutes, P1 and P2 conjugates were sterile-filtered through syringe filters with a pore size of 800 nm and warmed to 37°C before measurements. Raw distribution data was plotted in Graphpad Prism software and fit using a Gaussian curve, with the mean being taken as the particle size for that replicate. The average of three separate replicates was taken to find the mean particle size ± standard error of the mean (SEM). We also determined the zeta potential of the particles using a 90Plus Zeta Potential Analyzer (Brookhaven Instruments, Holtsville, NY). Particle formulations were dissolved in DI water at 1:20 v/v and warmed to 37°C before analysis. The average of three separate replicates was taken to find the mean zeta potential ± SEM.

Transmission electron microscopy (TEM) was performed to visualize P1 and P2 particles. Briefly, P1 or P2 particles were diluted in DI water 1:10 v/v and vortexed vigorously before being placed on a 200-mesh carbon-coated copper TEM grid that was previously cleaned by glow discharge. After 2 minutes, excess particle solution was blotted off and 1% uranyl acetate was added to the grid and allowed to sit for 2 minutes before being blotted dry. Images were collected on a JEOL JEM 1400 TEM.

### Cell culture

HepG2 cells (directly acquired from ATCC, Manassas, VA; Catalogue #HB-8065) were cultured in T-75 flasks using MEM supplemented with 10% FBS, 1% antibiotic-antimycotic, 1% sodium pyruvate, 1% non-essential amino acids, and 1 mL gentamicin. The cells were maintained at 37°C, 5% CO_2_, and 95% relative humidity and medium was changed every 48 hours. Cells were passaged at 80–90% confluency using a 0.25% trypsin/0.20% EDTA solution.

Cryopreserved vials of iCell^2^™ human cardiac myocytes (CMs) were obtained from Cellular Dynamics International, Inc (Madison, WI). iCell^2^™ CMs are highly purified (>98%) human induced pluripotent stem cell (hiPSC) derived cells that are cryopreserved after 30–31 days of cardiac directed differentiation. Cells were thawed and subsequently plated on Matrigel (500 μg/mL; BD Biosciences) coated costar 96 well plates (Corning) at a density of 50,000 cells per monolayer in differentiation medium essentially as described before.[28] Briefly, 100μL droplet of cells was plated in each well of a 96 well plate. Differentiation medium (EB20) consisting of 80% DMEM/F12, 0.1 mmol/L nonessential amino acids, 1 mmol/L l-glutamine, 0.1 mmol/L β-mercaptoethanol, 20% FBS, and 10μmol/L blebbistatin was used to culture the cells to promote attachment and monolayer formation. After 2 days, the media was switched to RPMI (Life Technologies) supplemented with B27 (Life Technologies). The cells were subsequently cultured for 5 more days at 37°C, in 5% CO2 and all displayed confluence and pacemaker activity before application of test reagents and subsequent cytotoxicity analysis. In total the cells were cultured for 7 days following thaw prior to application of treatments.

### Development of ectopic HepG2 tumor model

All animal procedures described in this work were reviewed and approved by the Institutional Animal Care and Use Committee (IACUC) at the University of Michigan. Mice were kept in specific pathogen-free (SPF) housing and were provided water and a regular diet ad libitum. As required by IACUC, animals were euthanized if tumors reached 2 cm in any dimension.

Male and female NSG mice (Jackson Laboratory, Bar Harbor, ME, USA) (4–10 weeks old) were used to prepare ectopic HepG2 tumor models in the flank. Briefly, 24 hours before tumor inoculation the mice were injected with cyclophosphamide (100 mg/kg) i.p. in order to suppress the reserve immune system and prevent tumor cell rejection. The next day, 2.5 x 10^6^ HepG2 cells were isolated and prepared 1:1 v:v in medium to Matrigel (Dow Corning, Midland, MI, USA) and kept on ice until the time of injection to prevent gelation of the Matrigel. We injected the cell suspension (150 μL volume) subcutaneously in the right flank of each mouse and placed a cotton swab on the site of injection after removal of the syringe to prevent leakage of cells. We monitored the health of the mice daily and measured the size of the tumor externally using digital calipers. The tumors were deemed ready for experiments once the tumor mass reached 50–100 mm^3^ (approximately 4–6 weeks).

### Intratumoral injection of P1 and P2 particles

Mice bearing ectopic tumors in the flank (50–100 mm^3^) were divided randomly into n = 5 per group for saline control, P1, P2, or free DOX treatments. Mice were anesthetized at 4–5% isoflurane for induction and reduced to 2–3% for maintenance during the procedure, with the carrier gas being oxygen. Anesthetized mice were given 0.5 mg/kg injections of free DOX or DOX-equivalent particle solutions in a final volume of 100 μL i.t. every 12 hours for 21 days using 27G insulin syringes (cumulative daily dose of DOX = 1 mg/kg). The injection site within the tumor was altered for each injection to create uniform distribution of the drug, and a cotton swab was placed on the injection site immediately upon removal of the syringe to prevent leakage of the drug/particles. Every other day, we measured body weight of the mice (data not shown) and used digital calipers to measure tumor volume using the formula $V\;({mm}^{3})=\frac{1}{2}LW^{2}$, where *L* and *W* are the longest and shortest diameters of the tumor, respectively. We normalized each tumor to its own volume at day 0 to account for differences in starting tumor volume. Results are presented as the mean percentage change in tumor volume every 2 days over the treatment period ± SEM. Two-way ANOVA with Tukey’s multiple comparison was used to determine statistical significance between the saline group compared to free DOX (*), P1 (#), and P2 ($) at each timepoint, and is denoted by *, #, or $ for p<0.05, **, ##, or $ $ for p<0.01, and ***, ###, or $ $ $ for p<0.001. Linear regression was used to determine the best-fit slopes of tumor growth during three periods: **Period I**, during treatment (day 0 to 21); **Period II,** after treatment (day 21 to 30); **Period III,** throughout the entire course of treatment (day 0 to 30). For **Period III** we also individually compared the best-fit slopes between treatments to test for significant differences between each group. There were no unexpected deaths that occurred before the end of the experiment.

### P1 and P2 particle toxicity against cardiomyocytes

The toxicity of P1, P2, and free DOX treatments on hiPSC CMs was measured using the change in confluency of CM monolayers, induction of apoptosis, and change in CM electrophysiology. For the confluency measurements, either RPMI/B27 blank medium (negative control), 0.1% Triton X-100 (positive control), or P1/P2/free DOX treatments at a DOX-equivalent range of 1–100 nM were dissolved in RPMI/B27 and incubated with CMs for 32 hours. The monolayers were imaged using the phase-contrast channel of an IncuCyte time-lapse microscope (Essen Biosciences, Ann Arbor, MI), and IncuCyte Zoom software was used to create a confluency mask to quantify the amount of monolayer remaining as a percentage of the total well surface area. Each well was checked for 100% confluency before treatment, and then imaged every 4 hours at 4x magnification. The decrease in confluency for each well from the start of the experiment was calculated and then normalized to the decrease induced by 0.1% Triton X-100 (positive control) to determine the relative % toxicity of P1, P2, and free DOX treatments. Toxicity was investigated for five replicates and results are reported as the mean ± SEM. A Student’s t-test was used to identify treatments that caused a statistically higher toxicity than the blank medium (negative control), with significance indicated by *** for p<0.001.

For apoptosis screening, hiPSC CMs were again cultured for 7 days at 37°C, 5% CO2 and checked for display of spontaneous pacemaker activity. The cells were treated with RPMI/B27 (Life Technologies) supplemented with IncuCyte Annexin V Green Reagent for Apoptosis (1:100) (Essen Biosciences) and either blank medium (negative control), P1, P2, or free DOX at 100 nM DOX-equivalent concentration. Using the phase-contrast and green fluorescent channels of the microscope, the plates were imaged every 4 hours for a total of 24 hours. Induction of apoptosis was measured using IncuCyte Zoom software, and results are reported as the mean of five replicates ± SEM. Student’s t-tests were performed to measure the statistical difference between P1, P2, or free DOX treatments from the negative control and are denoted by *** for p<0.001.

To measure the change in electrophysiology of treated CMs, optical action potentials were recorded using a FluoVolt™ membrane potential probe (Life Technologies, F10488) after the apoptosis assay. The probe was incubated with the monolayers for 30 minutes and then removed with a wash of Hanks balanced salt solution (HBSS, Life Technologies) for an additional 30 minutes before optical mapping recordings. The spontaneous action potentials were recorded using a CCD camera (Red-Shirt Little Joe, Scimeasure, Decatur, GA, 200 fps, 80×80 pixels) with the appropriate emission filters and LED illumination.[28] 10 second movie recordings were filtered in both the time and space domains and analysis was done using custom software (Scroll, written by Dr. Sergey Mironov). The number of monolayers displaying normal or abnormal rhythm was quantified for each test condition.

### Magnetic resonance imaging (MRI) to assess cardiac function

Healthy, male and female NSG mice (6–10 weeks old) were randomly assigned to one of four treatment groups (saline control, P1, P2, or free DOX; n = 3 per group). Personnel acquiring the MRI data (A.C.C.) were blinded to treatment groups. Before treatment, the mice underwent MR imaging as described above to assess baseline cardiac function (t = Week 0). The mice were then given i.p. injections of either P1, P2, or free DOX at 1 mg/kg DOX-equivalent dosing every 2 days for 21 days. The mice were imaged via MR once a week during treatment (t = Weeks 1, 2, and 3), and one final time one week after the last injection (t = Week 4).

Mice were anesthetized with 1.25–2% isoflurane in 1 L/min of oxygen. Animals were then placed in the supine position and imaged at 7T using a Direct Drive console (Agilent Technologies, Santa Clara, CA) and a 40 mm inner diameter transmit-receive volume coil (Morris Instruments, Ontario, Canada). Core temperature was controlled to 37°C within +/- 0.2°C using a custom-built PID controller (Labview, National Instruments, Austin TX) interfaced with a commercially available small animal system which includes a heater blowing warm air and a rectal temperature probe (SA Instruments, Stony Brook, NY). Respiration and ECG were also monitored.

Coronal 2D acquisitions were used to plan the long axis slices of the heart. Long axis acquisitions of the heart at end-diastole and end-systole were obtained for each mouse undergoing treatment. Five to six 2D contiguous slices were planned through the heart depending on the size of the organ. For each slice, a cardiac-gated and respiratory compensated 2D CINE acquisition with 12 frames was performed [TR/TE 180/2 ms, FOV (30 mm)^2^, □□30^o^, matrix 128^2^ zero-filled to 256^2^, slice thickness 1 mm, NEX 4, resolution (117μm)^2^]. The endocardial area of each frame was defined manually using Analyze. For each slice, the end-diastolic and end-systolic areas were determined by selecting the maximum and minimum areas, respectively. The left ventricle end-diastolic volume (LVEDV), left ventricle end-systolic volume (LVESV), stroke volume (SV), and cardiac output (CO) were calculated by using Eqs 1–4:

$$LVEDV\;(\mu L)=\sum_{i=1}^{5-6}{Slice\;area\;max}_{i}*Slice\;thickness$$ (1)

$$LVESV\;(\mu L)=\sum_{i=1}^{5-6}{Slice\;area\;min}_{i}*Slice\;thickness$$ (2)

$$SV\;\left(\frac{\mu L}{beat}\right)=LVEDV-LVESV$$ (3)

$$CO\;(\frac{mL}{min})=SV*heart\;rate$$ (4)

Results are presented as the mean of each treatment group ± SEM. Two-way ANOVA with Tukey’s multiple comparisons test was used to test the statistical significance between P1, P2, and free DOX compared to the saline group, except for the free DOX group at Week 2, where a t-test was used because replicate numbers did not match due to death (n = 1) in the free DOX group. Significance is denoted by * for p<0.05 and ** for p<0.01.

## Results and discussion

### Synthesis of P1 and P2 particles is reproducible

We[24–27,29] and others[30–33] have previously discussed the utility of using G5 PAMAM dendrimers as targeted drug delivery vehicles. G5 PAMAM dendrimers exhibit a unique combination of properties that give them advantages over other nanocarrier options, such as their high water solubility, biocompatibility, controllable synthesis, and size/weight properties that extend circulation time and allow penetration into tumor tissue. Further, the branched architecture and high density of functional groups at the G5 surface enable the facile loading of multiple payloads, which is crucial for our work to simultaneously load targeting agents (e.g. NAcGal_β_) and enzyme-sensitive DOX linkages (e.g. L3- or L4-DOX).

We functionalized G5 dendrimers with NAcGal_β_-PEG*c* targeting branches as well as L3-DOX or L4-DOX linkages using our previously reported synthetic strategies.[27] We first conjugated 16.2 NAcGal_β_-PEG*c* units onto the G5 surface, as confirmed by NMR and MALDI spectra (**S1 Fig**). This corresponds to 12.7 mole% PEGylation, which facilitates the PEG molecules to adopt a “brush” conformation. PEG in the brush conformation completely covers the G5 surface, preventing protein adsorption that would lead to clearance of the particles by the reticuloendothelial system (RES).[27,34,35] Next, we conjugated L3-DOX or L4-DOX linkages onto PEGylated G5 using click chemistry[26,27] to achieve P1 and P2 particles (**S2 and S3 Figs**). We attained similar loading of DOX on P1 (compound **12**) [16.2(NAcGal_β_-PEG*c*)-G5-(L3-DOX)13.1] and P2 (compound **13**) [16.2(NAcGal_β_-PEG*c*)-G5-(L4-DOX)13.4], with 13.1 and 13.4 moles of DOX per G5, respectively (**Table 1**). As prepared, P1 and P2 particles maintained aqueous solubility at concentrations up to 1.30 mg/mL. Given the similarity in PEGylation, NAcGal-, and DOX-loading to our previously reported particles,[25–27] it is important to highlight the batch-to-batch reproducibility of our system. The robust chemical practices we use allow the repeated synthesis of P1 and P2 particles with minimal variability, offering a strong advantage over other molecular therapeutics that are difficult to scale up due to batch-to-batch variation.[36]

**Table 1:** Physicochemical properties of P1 and P2 particles.

Particle Name	Chemical Composition	MW (Da)	Particle Size (nm)	Zeta Potential (mV)
**P1**	(NAcGal_β_-PEG*c*)_16.6_-G5-(L3-DOX)_11.9_	82,577	7.00 ± 0.31	-0.63 ± 0.28
**P2**	(NAcGal_β_-PEG*c*)_16.6_-G5-(L4-DOX)_13.4_	83,277	6.95 ± 0.17	-0.46 ± 0.23

P1 and P2 particles had molecular weights (MW) of 82,577 and 83,277 dalton (Da), respectively (**Table 1**). Dynamic light scattering (DLS) results show P1 and P2 particles had hydrodynamic diameters (HD) of 7.00 ± 0.31 nm and 6.95 ± 0.17 nm, respectively, (**Table 1; S7 Fig**). TEM analysis revealed individually resolvable particles with spherical morphology, which is expected given the spherical nature of PAMAM dendrimers.[37] Further, zeta potential measurements indicated that both particles had a neutral surface charge (**Table 1**). As we described previously,[27] these physicochemical properties ensure the particles will be able to evade renal filtration and clearance by the reticuloendothelial system (RES). More importantly, they allow P1 and P2 particles to exploit the enhanced permeability and retention (EPR) effect,[38,39] which allows the particles to extravasate into the tumor interstitium through leaky vasculature and be retained there due to lack of a proper lymphatic drainage system.

### Tumor model development

The combination of immune-compromised NSG mice with an immunosuppressant given prior to HepG2 inoculation resulted in 100% tumor take in injected mice. By the third week after injection, tumors became palpable and volumes were measured at 23.8 ± 1.9, 50.3 ± 2.7, and 86.4 ± 2.7 mm^3^ at weeks 3, 4, and 5, respectively. As soon as a tumor volume reached 50–100 mm^3^, mice were randomly divided into a treatment group. The average tumor volume at day 0 was 58.9 ± 4.8 mm^3^ and there were no statistically significant differences between any of the groups.

### P1 and P2 particles inhibit tumor growth earlier and to a greater degree than free DOX

**Fig 3:**
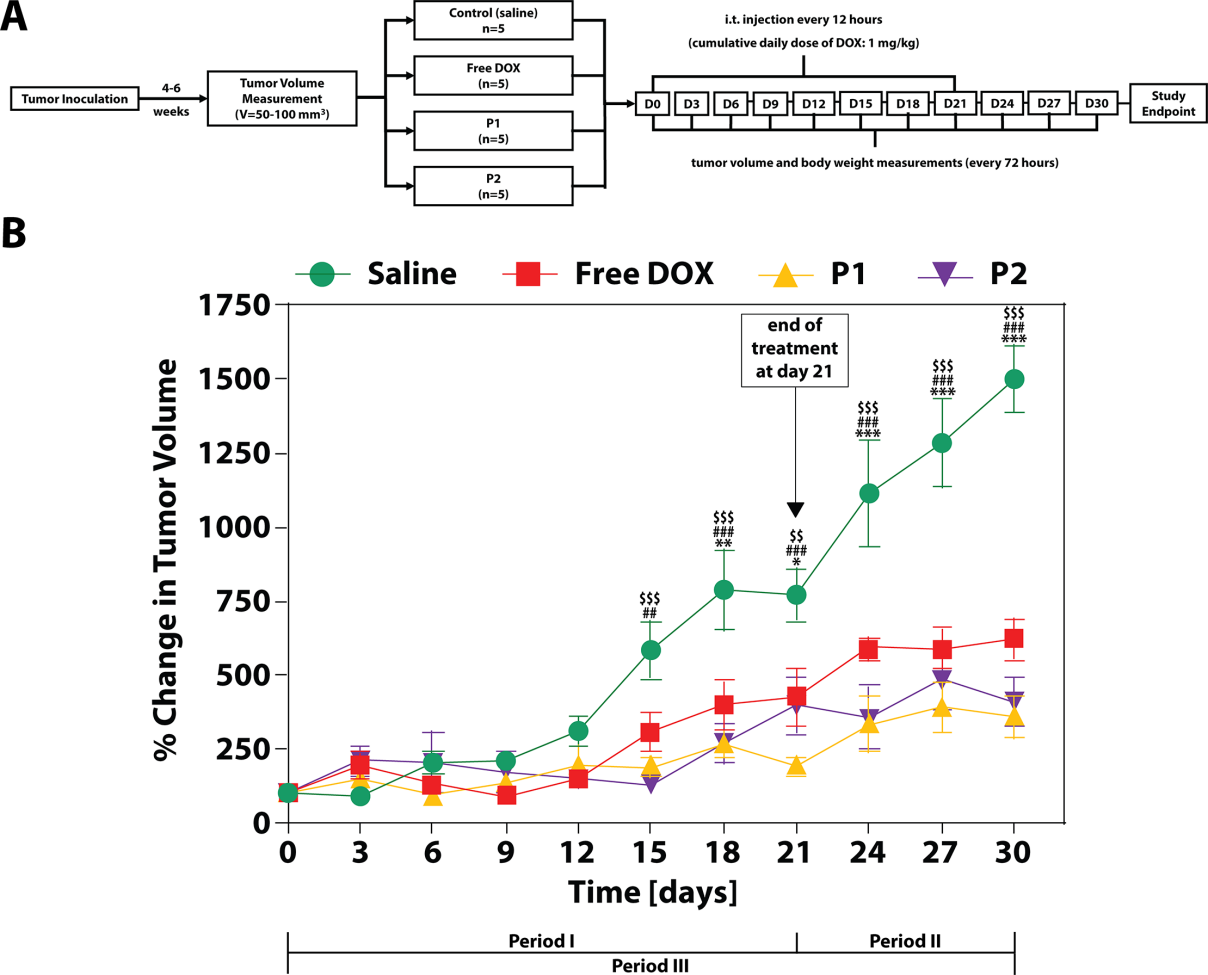
Intratumoral delivery of P1 and P2 particles achieves antitumor activity comparable to free DOX. We measured the antitumor activity of P1 and P2 particles after intratumoral (i.t.) delivery to ectopic HepG2 tumors developed in nod scid gamma (NSG) mice, in order to mimic the clinical delivery of DOX through hepatic arterial infusion. **(A)** When tumors reached 50–100 mm^3^ in volume, animals were randomly divided into treatment groups of either saline, P1, P2, or free DOX, with each group receiving a DOX-equivalent dose of 1 mg/kg injected twice daily for 21 days. We monitored tumor volume every 3 days through day 30, and normalized tumor volumes to their starting volume at day 0. **(B)** Results show that saline-treated tumors reached a change in tumor volume of 1501 ± 115% by day 30, while P1, P2, and free DOX treatments inhibited tumor growth by 76.1%, 72.9%, and 58.6%, respectively, compared to the saline controls. Linear regression results show that while free DOX inhibits tumor growth by 2.5-fold over the entire treatment period, P1 and P2 particles inhibit growth 5.1- and 4.4-fold, respectively. Results are presented as a mean of five replicates ± standard error of the mean (SEM). Two-way ANOVA was used to determine statistical significance between the saline group compared to free DOX (*), P1 (#), or P2 ($) at each timepoint, and is denoted by *, #, or $ for p<0.05, **, ##, or $ $ for p<0.01, and ***, ###, or $ $ $ for p<0.001.

We were interested to see if P1 and P2 particles would exhibit antitumor activity comparable to free DOX in NSG mice bearing ectopic HepG2 tumors. Therefore, we measured the effect of i.t. injection of either P1, P2, or free DOX at an equivalent DOX dosage of 1 mg/kg per day for 21 days, measuring tumor volume by digital caliper. It is important to note that the clinical dosage of DOX conventionally used is between 60–75 mg/m^2^,[40–42] which equates to 1.6–2.0 mg/kg using accepted dose conversion methods for humans.[43] Therefore, we tested a dose that is 37.5 to 50% lower than clinical doses and looked for a statistically significant inhibition of tumor growth as the key outcome to measure therapeutic efficacy of treatment, in comparison to saline-treated tumors (**Fig 3**). As such, P1-, P2-, and free DOX-treated tumors were not statistically different in tumor volume compared to saline-treated tumors through day 12 of treatment. After day 12, tumor growth was greater for the saline group than for any of the treatment groups. P1 and P2 particles induced an earlier onset of therapeutic activity, as seen by statistically significant inhibition of tumor growth compared to the saline control by day 15 (p<0.01, saline vs. P1; p<0.001, saline vs. P2; p = 0.08, saline vs. DOX). At day 18, however, all three treatment groups reached statistically significant tumor growth inhibition, although the effect from P1 and P2 particles had stronger significance (p<0.0001, saline vs. P1 or P2; p = 0.01, saline vs. free DOX). By the end of treatment at day 21, the mean change in tumor volume (MCTV) for saline-treated tumors was 771.1 ± 87.3%, while for the P1, P2, and free DOX groups it was 190.7 ± 35.4, 398.6 ± 98.7, and 424.4 ± 100.6%, respectively. Therefore, the treatments inhibited tumor growth by 75.3, 48.3, and 45.0%, respectively (p<0.0001, saline vs. P1; p<0.01, saline vs. P2; p<0.05, saline vs. free DOX). Representative images of tumors at day 21 can be seen in **S9 Fig**. Upon the removal of treatment, saline-treated tumors continued to experience large growth rates, reaching an MCTV of 1501 ± 115%, while P1-, P2-, and free DOX-treated tumors reached 358.8 ± 71.9, 406.8 ± 85.9, and 622.0 ± 70.0%, respectively. This equated to a 76.1, 72.9, and 58.6% reduction in tumor growth by the respective treatments at day 30 (p<0.0001 for P1, P2, and DOX vs. saline). It is important to note that 100% of animals survived through day 30. The lack of toxicity from free DOX is likely due to the low dosage administered as well as the lymphatic clearance of subcutaneously-injected compounds (versus systemic distribution).[44]

We compared the inhibitory rates of P1, P2, and free DOX treatments during (**Period I**), after (**Period II**), and over the entire course of treatment (**Period III**) to identify the effect of presence or absence of treatment on antitumor activity between the groups. Results of the linear regression tests for **Periods I**, **II**, and **III** can be found in **Table 2**, with the slopes of each treatment group listed. During **Period I**, slopes of P1, P2, and free DOX treatment groups are 6.0-, 4.4, and 2.4-fold smaller than that of the saline treatment group, with strong statistical difference between all groups. This indicates that not only were the treatments effective at tumor growth inhibition, but P1 and P2 exhibit stronger effects over free DOX. After the removal of treatment (**Period II**), tumor growth of the saline group increased substantially, indicated by a 2.1-fold increase compared to **Period I**. We hypothesize that the removal of injections using 27G needles improved the growth conditions of the tumor, particularly by allowing it to restore and maintain a high interstitial fluid pressure that is known to promote tumor cell proliferation.[45] During this stage, slopes of P1 and free DOX treatment groups increased by 3.0 and 1.3-fold, respectively, suggesting that their activity was decreased upon treatment cessation and this effect was the largest for P1 particles. Interestingly, the slope of the P2 group reduced 1.6-folds compared to **Period I**. This suggests that P2 particles may have either a time-delay in initiating activity or a prolonged effect after ending treatment. Over the entire study (**Period III**), the slopes of P1, P2, and free DOX are different from the saline group with strong significance (p<0.0001 for all). While the slope of the free DOX group is 2.5-fold smaller than that of the saline group, P1 and P2 particles have stronger inhibitory effects, slowing tumor growth by 5.1- and 4.4-fold, respectively (**Table 2**).

**Table 2:** Linear regression results of tumor growth.

Period	Time Period	Slope			
		Saline	DOX	P1	P2
**I**	Day 0 to 21	37.4 ± 5.1	15.4 ± 4.1	6.2 ± 1.9	8.5 ± 4.0
**II**	Day 21 to 30	78.7 ± 8.5	19.9 ± 8.2	18.9 ± 9.1	5.2 ± 9.3
**III**	Day 0 to 30	48.9 ± 4.0	19.7 ± 2.4	9.6 ± 1.4	11.2 ± 2.3

The therapeutic advantage of P1 and P2 over free DOX is exemplified by the strong significance when comparing the slopes between the respective groups over **Period III** (p = 0.0021, P1 vs. free DOX; p = 0.0212, P2 vs. free DOX). This advantage is surprising given that our *in vitro* cytotoxicity results showed free DOX concentrations required to inhibit 50% of tumor cell growth (i.e. IC_50_) was approximately 57- and 10-folds lower than P1 and P2, respectively.[27]

However, in tumor tissue, P1/P2 particles exhibited a therapeutic advantage over free DOX (**Fig 3**). We hypothesize that the EPR effect sequesters the nanoparticles within tumor tissue due to the lack of a proper lymphatic drainage system.[46] Small molecules like free DOX (MW: 542 Da), however, can diffuse out of the tumor via capillaries in order to reach equilibrium throughout the body,[47] thereby decreasing the intratumoral DOX concentration and the antitumor activity it causes.

### Potential differences in tumor growth inhibition between P1 and P2 particles

In our previous work,[27] P2 particles exhibited a 5.9-fold higher cytotoxic activity than P1 *in vitro*, which we explained was due to the more labile linkage chemistry (e.g. L4) attaching DOX to the G5 surface. Our metabolomics analysis showed that two active DOX molecules released from both P1 and P2 –isomers of tetracenomycin (TCM), either F1 methylester or D1 –were in higher abundance intracellularly from P1 particles than those released from P2. Conversely, the extracellular abundance, particularly of TCM D1 metabolites, was higher for P2-treated cells compared to P1. We explained this phenomenon by considering the release kinetics of P1 vs. P2. We estimated that the lower intracellular concentrations of TCM molecules in P2-treated cells was a result of the quicker release (due to the more labile linkage chemistry), which subsequently allowed clearance from the tumor cells into the extracellular media. Despite the efflux, we hypothesized that the earlier release of TCM molecules led to higher initial intracellular concentrations and the higher observed toxicity.[27]

Interestingly, the differences in slope of tumor growth between P1 and P2 over the entire treatment period, while not statistically significant (p = 0.057), suggest that P1 particles may have an advantage in antitumor activity over P2 particles (**Fig 3**, **Table 2**). We explain this potential difference by considering the changes in the microenvironment between *in vitro* cell culture and an *in vivo* tumor model. In our *in vitro* cytotoxicity assays,[27] after inducing toxicity in the host tumor cell, TCM D1 molecules from P2-treated cells were effluxed into the extracellular medium, where they would reside and potentially be internalized by other cells in culture via passive diffusion. We hypothesize that this cyclic shuffling between cells is what led to the increased toxicity in the midst of P2 particles, while for P1-treated cells, the released TCM D1 molecules were sequestered intracellularly and able to induce apoptosis in a smaller percentage of cells. However, in tumor tissue the same lymphatic and vascular drainage that reduces free DOX toxicity may also clear TCM D1 molecules once effluxed from treated cells due to its small size (MW: 336 Da). In other words, if P2 particles do in fact exhibit reduced activity compared to P1, it may be because the cycling of TCM molecules from cell to cell is not as prominent *in vivo* due to drainage of the small molecule from tumor tissue. Contrarily, the intracellular sequestration of P1-derived TCM D1 molecules may allow for sustained therapeutic activity within the tumor, conferring the higher therapeutic activity observed for P1 particles. Further studies including metabolomics analysis of treated tumor tissue to measure the intratumoral and intracellular concentrations of P1- and P2-derived DOX metabolites will help elucidate the qualitative therapeutic differences between P1 and P2 particles observed here.

### P1 and P2 particles do not induce toxicity in cardiomyocytes

**Fig 4:**
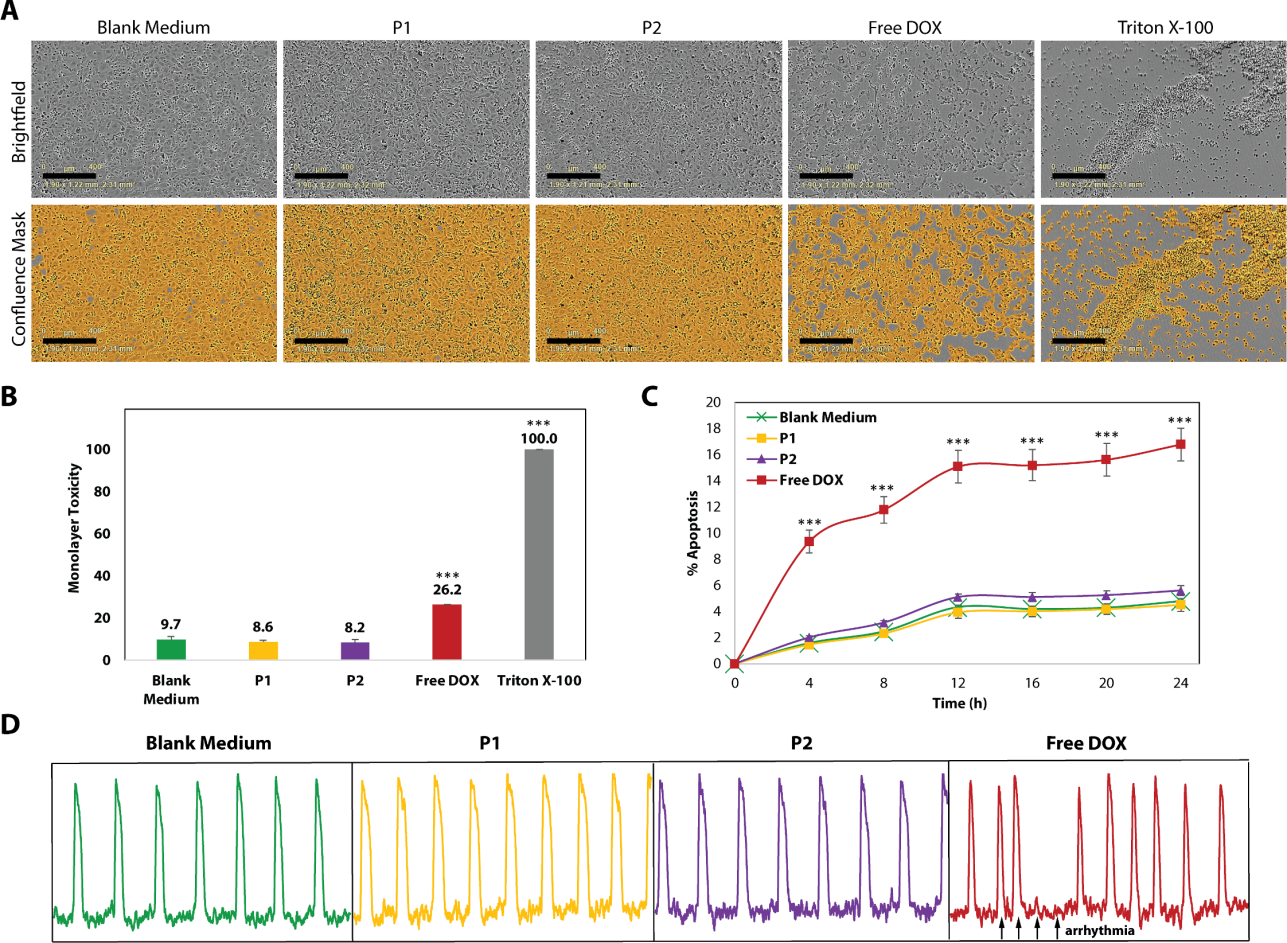
P1 and P2 particles avoid DOX toxicity towards cardiomyocytes. The effect of P1 and P2 particles on cardiomyocyte (CM) viability and electrophysiological behavior was investigated in comparison to free DOX. **(A)** Human induced pluripotent stem cell-derived CMs were treated with P1, P2, or free DOX at 100 nM DOX and imaged at 4X magnification for 32 hours. Images show the CMs remaining on the surface at the end of treatment in the top row, and a confluence mask that was created to quantitatively measure the surface area coverage in the bottom row. Scale bars represent 400 μm. **(B)** Quantitation of the toxicity towards CMs as a measure of the decrease in confluence using the aforementioned mask, normalized to the positive control of Triton X-100. P1 and P2 particles exhibit normal (8–9%) toxicity compared to the negative control (blank medium), while free DOX induces 26.2 ± 0.2% toxicity. **(C)** Using Annexin V-labeling to detect CMs undergoing early apoptosis over a 24 hour treatment period, P1- and P2-treated CMs exhibit no more induction of apoptosis than the negative control, while free DOX treatments induce up to 16.8 ± 1.2% apoptosis by 24 hours. **(D)** Treated CMs were monitored for spontaneous action potentials using a FluoVolt membrane potential probe. While CMs treated by blank medium, P1, and P2 exhibited no signs of arrhythmia, 33% of DOX-treated CMs expressed arrhythmic beating (black arrows). Images represent a timescale of 10s. Results in Panel **B** and **C** are represented as the mean of five replicates ± SEM. A Student’s t-test was used to compare P1, P2, free DOX, or Triton X-100 treatments to the negative control, and statistical significance is denoted by *** for p<0.001.

We investigated the effect of P1 and P2 particles on human induced pluripotent stem cell-derived cardiomyocytes (hiPSC CMs) by measuring the viability of treated monolayers, presence of apoptotic markers, and the presence of arrhythmia in treated CMs. To measure monolayer viability, we treated CMs with either P1, P2, or free DOX at a DOX-equivalent dosage of 100 nM for 32 hours, imaging each well at 4X magnification every 4 hours (**Fig 4A**). We also included blank medium and Triton X-100 as negative and positive controls, respectively. IncuCyte Zoom software was used to develop a confluence mask that quantitatively measured the total surface area covered by CMs at each timepoint (**Fig 4B**). After normalizing to the positive control at the end of treatment, P1- and P2-treated CMs do not experience any decrease in surface coverage besides for normal cell death (8–9%), which is comparable to the negative control (**Fig 4A & 4B**). Meanwhile, the free DOX-treated CMs experienced 26.2 ± 0.2% toxicity, which was statistically significant from the negative control (p<0.001, **Fig 4A & 4B**). This rate of toxicity towards CMs after an acute exposure to DOX is expected, given previous work in both primary and hiPSC CMs [26,48]. We further measured the number of CMs undergoing early apoptosis using an Annexin-V fluorescent stain during 24 hours of treatment with either blank medium, P1, P2, or free DOX at the same DOX dosage (**Fig 4C**). Results show that while P1- and P2-treated cells undergo normal (~5%) apoptosis compared to the negative control over 24 hours, free DOX induces apoptosis at a significantly higher rate, achieving 16.8 ± 1.2% apoptosis at the end of the treatment window (p<0.001 compared to the negative control). Finally, we monitored the electrophysiological behavior of treated CMs by tracing the action potential using a FluoroVolt membrane potential probe. Representative traces of the action potential over a 10s period can be seen in **Fig 4D**. Free DOX-treated CMs experienced either abnormal or absent beating in 33% of treated monolayers, which is expected for CMs being treated by DOX.[48] Contrarily, CMs treated by blank medium, P1, and P2 experienced no arrhythmia. These results collectively indicate that the toxicity, induction of apoptosis, and dysregulation of electrophysiological behavior associated with free DOX can be avoided by P1 and P2 particles, likely due to the lack of the ASGPR receptor on CMs as well as the enzyme-activated DOX linkages that prevent DOX release from P1/P2 within the CM cytoplasm.

### P1 and P2 particles maintain cardiac function in treated mice

**Fig 5:**
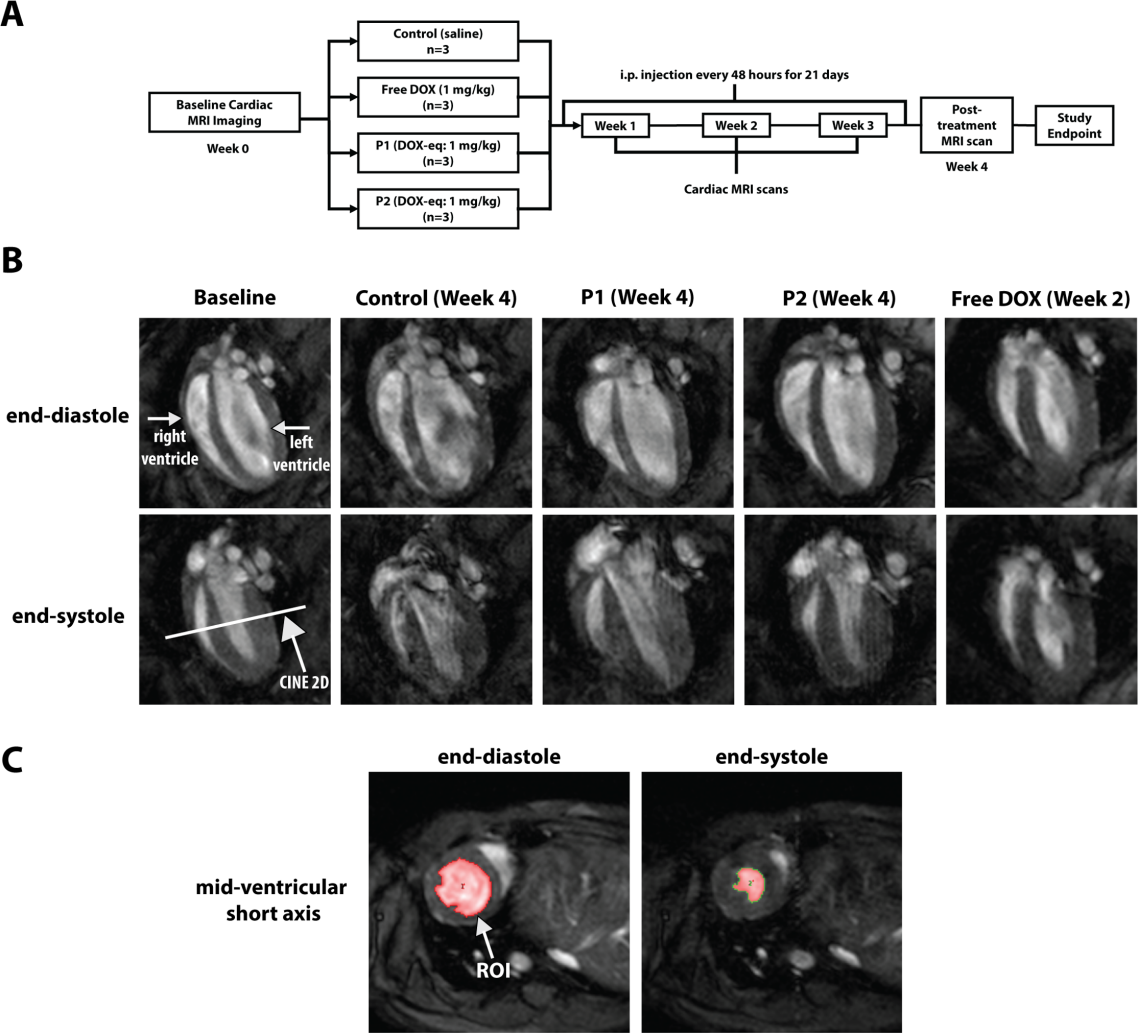
Measurement of cardiac function by MR imaging. We measured the cardiac function of mice undergoing treatment by saline, P1, P2, or free DOX. (**A**) Treatment and imaging regimen used. Healthy, NSG mice were randomly divided into four groups and assessed for their baseline cardiac function using magnetic resonance imaging (MRI). Starting at day 0, mice were given intraperitoneal (i.p.) injections of one of the four treatments every 48 hours for 21 days. We imaged the hearts weekly during treatment and also one week post-treatment. (**B**) Representative, long axis acquisitions at end-diastole and end-systole of mice treated by control, P1, P2, or free DOX at the final week of imaging possible. (**C**) For each 2D short-axis slice, a cardiac-gated and respiratory compensated 2D CINE acquisition with 12 frames was performed and the endocardial area of each frame defined using Analyze. The end-diastolic and end-systolic areas were found in each slice and are represented by the highlighted region of interest (ROI). LVEDV, LVESV, SV, and CO were then found using Eqs 1–4.

To evaluate the effect of particle treatment on the cardiac function of mice, we administered either saline, P1, P2, or free DOX at a DOX-equivalent dosage of 1 mg/kg (cumulative weekly dose of 3.5 mg/kg) i.p. and used weekly MR imaging of the heart to assess cardiac function (**Fig 5A**). Cardiac MR is a standard noninvasive practice to assess the effect of chemotherapy administration on cardiac function,[14,49–51] with decreases in LVEDV, LVESV, SV, and CO indicating cardiotoxicity after doxorubicin treatment, in humans and in mice.[50–52]

Saline, P1, and P2 groups showed no signs of morbidity through Week 4, and accordingly had a 100% survival rate. Qualitative visual assessment of the DOX group during treatment showed signs of unkempt fur, squinted eyes, and hunched posture within 1 week of beginning treatment. 100% of the free DOX group presented characteristics of palmar-plantar erythrodysesthesia (i.e. hand-foot syndrome), a common clinical side effect of DOX treatment,[53] by day 11. The median survival time for DOX-treated mice was 14 days, with complete group mortality by day 15 (**S10 Fig**). While this is a low survival rate, the rate falls into the large range of median survival (2–14 weeks) observed in similar studies administering a cumulative weekly DOX dosage of 3–4 mg/kg DOX.[54–56] Long axis 2D images of the hearts were obtained at both end-diastole and end-systole throughout the depth of the organ (**Fig 5B**). Qualitatively, hearts by one week post-treatment (Week 4) for the saline, P1, and P2 groups show no morphological changes compared to the same hearts at baseline. However, for the free DOX group, at the last possible imaging time (Week 2), distinct changes in ventricular morphology can be seen. Specifically, smaller ventricle size at diastole and larger ventricle size at systole, compared to the baseline and control. This represents lower contractility of the heart tissue, which is representative of damaged cardiac function.[57–59]

**Fig 6:**
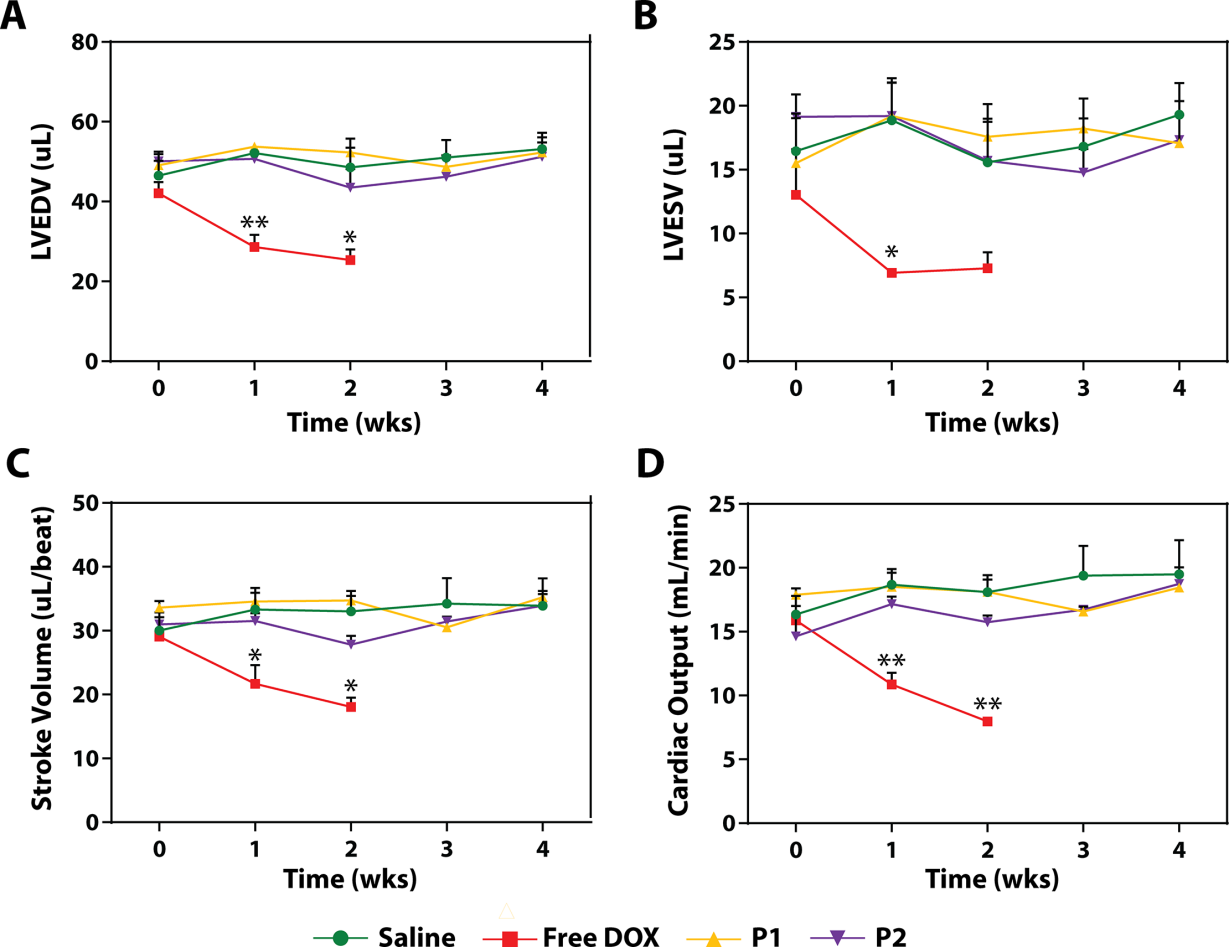
P1- and P2-mediated delivery of DOX escapes cardiotoxicity associated with free DOX administration. We measured the cardiac function of mice undergoing treatment by saline, P1, P2, or free DOX. Results show the effects of each treatment on the (**A**) left ventricular end diastolic volume (LVEDV), (**B**) left ventricular end systolic volume (LVESV), (**C**) stroke volume (SV = LVEDV–LVESV), and (**D**) cardiac output (SV*heart rate). The statistically significant decrease in cardiac function by all four metrics is obvious already after Week 1 of free DOX treatment, while P1 and P2 particles do not induce any toxicity compared to the control. Further, free DOX mice had a 100% death rate by day 15, while P1- and P2-treated had a 100% survival rate during the monitoring period. Results are presented as a mean of three replicates ± SEM. Two-way ANOVA was used to test the statistical significance between P1, P2, and free DOX compared to the saline group, except for the free DOX group at Week 2, where a t-test was used because mice numbers did not match due to death in the free DOX group. Significance is denoted by * for p<0.05 and ** for p<0.01.

We calculated the LVEDV, LVESV, SV, and CO values using maximal and minimal endocardial areas at diastole and systole, respectively, using 2D CINE acquisitions of each 2D long-axis slice acquired (**Fig 5C**). Mean LVEDV, LVESV, SV, and CO values at baseline between all animals were 46.9 ± 1.6 μL, 16.0 ± 1.5 μL, 30.9 ± 0.8 μL/beat, and 16.2 ± 0.7 mL/min, respectively. These values are normal for mice[60,61] and were similar between each treatment group (**Fig 6A–6D)**. By the end of the monitoring period (Week 4), the control group of mice exhibited no decrease in LVEDV, LVESV, SV, nor CO at Week 4 compared to baseline (p>>0.05 for all values). Similarly, P1 and P2 particles induced no changes in either LVEDV, LVESV, SV, or CO between their baseline and any timepoint (p>>0.05). There were also no differences between P1 and P2 groups at any timepoint (p>>0.05). For free DOX-treated mice, however, there was an obvious and significant decrease in cardiac function that is noticeable within the first week of treatment. By Week 2 (the last possible imaging time for the free DOX group), LVEDV, SV, and CO dropped by 39.8, 37.9, and 49.8%, respectively, to 25.3 ± 2.7 μL, 18.1 ± 1.5 μL/beat, and 8.0 ± 0.2 mL/min (p<0.05, p<0.05, and p<0.001; **Fig 6A, 6C and 6D**). Heart rate (HR) values were statistically not different from each other between all groups, allowing us to conclude that changes in CO (SV*HR) were solely dependent on changes in SV. While there was no statistical significance between LVESV at baseline compared to Week 2 (7.3 ± 1.2 μL, p = 0.2) (**Fig 6B**), there was a 44.1% decrease observed, suggesting that the end systolic volume was also affected by free DOX treatment.

Our data supports the hypothesis that the combination of NAcGal-mediated targeting, PEGylation (which is known to limit distribution to off-target organs[62,63]), and a particle size profile that disables free diffusion across intact vasculature[64] is able to spare P1 and P2 particles from cardiac distribution. Further, we have previously shown that the enzyme-dependent release of DOX from L3-DOX and L4-DOX linkages does not occur in cardiomyocytes, due to the enzymes being solely of hepatic origin,[65–67] and as such we saw no resulting toxicity towards cardiomyocytes.[26] This suggests that even if P1/P2 particles were to distribute to heart tissue *in vivo*, DOX release would not occur and therefore no decrease in cardiac function should be observed. A recent report by Zhang *et al*. established an MR protocol to assess DOX-induced cardiotoxicity after weekly IP injections of DOX,[14] and we followed a similar protocol to assess P1-, P2-, or free DOX-induced toxicity in the present work. We chose not to assess the cardiotoxicity of mice undergoing i.t. therapy of ectopic tumors because it is well known that the clearance of subcutaneously-injected compounds depends on a complex interplay between the vascular network, lymphatic capillaries, and the hypodermal interstitium. In particular, for molecules larger than 20 kDa, diffusion into blood capillaries is inhibited and the lymphatic system dominates clearance.[68] We predicted therefore that P1 and P2 particles would not achieve ample concentrations in the systemic circulation that are required to induce a representative cardiotoxic effect. Alternatively, given that i.p.-injected compounds are primarily cleared through the portal vein,[69] i.p. injection of P1 and P2 particles are a better predictor of cardiotoxicity given that they would have direct localization in the systemic circulation. Our results showing the maintenance of cardiac function after treatment suggest that P1 and P2 particles are equipped to avoid distribution to heart tissue and/or prevent release of DOX within the heart. Further studies using metabolomics and biodistribution analysis will help identify whether it is the HCC-specific targeting or the enzyme-sensitive DOX release, or a combination of both, that confers the cardio-protective effect of P1/P2 particles.

Efforts to package DOX in a way that would improve therapeutic efficacy but minimize its cardiac distribution and toxicity have been made before. The use of liposomal DOX formulations, such as DOXIL or CAELYX, have proven their non-inferiority to DOX in therapeutic efficacy while also minimizing the occurrence of cardiotoxicity in patients with multiple myeloma[23] and metastatic breast cancer.[41] The decrease in toxicity has been credited to the ability of liposomes to prevent DOX diffusion through vascular junctions,[23] as well as the ability of PEGylation to increase circulation half-life and thereby decrease off-target tissue distribution.[41] Unfortunately, the extension of these novel DOX formulations has not seen the same clinical success when applied to HCC therapy. While patients were spared from cardiotoxicity, several clinical trials measuring the effect of liposomal DOX exhibited minimal therapeutic efficacy compared to DOX alone.[70–72] Valle *et al*. described a 0% response rate to PEGylated liposomal DOX for HCC patients, and in fact the trial was ended early because it did not reach the minimal threshold of activity at initial checkpoints.[70] While the minimal therapeutic efficacy has yet to be mechanistically explained, it is postulated that the increased circulation time of liposomal DOX also prevents significant uptake into hepatic tissue, and without specific molecular targeting to HCC, the formulation is left with minute concentrations in hepatic tumor tissue.[23,70] Our targeting strategy, however, equips P1/P2 to overcome the minimal distribution to hepatic tumor tissue observed with its liposomal counterparts.

The P1/P2 formulation could replace DOX in a multitude of procedures that it is used in currently. First and foremost, given that i.t. therapy is a good predictor of intra-arterial efficacy,[73] the P1/P2 formulation has high potential to be a viable replacement for free DOX in HAI procedures. Further, with the recent successes of combinatorial treatment involving chemotherapy cocktails, surgery, and/or targeted molecular therapies like sorafenib,[74–77] P1/P2 particles could be an integral part of the new era of advanced HCC therapy. Sorafenib, due to its modest effect on survival benefit of HCC patients when delivered alone,[74,78] has been combined with DOX (administered either systemically[75] or through TACE[76,77]) and shown improved therapy over both agents given alone.[76,77,79] However, patients experienced the expected DOX toxicities such as cardiotoxicity, myelosuppression, hand-foot syndrome, and neutropenia. In another interesting approach, DOX delivered by TACE is used as a predecessor to surgery in order to downsize the tumor.[80] Not surprisingly, DOX morbidities developed for these patients as well. In both cases, P1/P2 particles offer an alternative DOX formulation that can either be used in combination with sorafenib or simply to reduce a large tumor before removing it surgically, potentially mitigating the observed toxicities while achieving comparable (if not better) therapeutic results. Another recent study showed that HAI of epirubicin and cisplatin combined with i.t. delivery of interferon-gamma and 5-fluorouacil achieved a complete response in 66% of patients with advanced HCC.[74] Despite this success, however, morbidities of myelosuppression and flu-like symptoms developed, likely due to the toxicities from one or several of the chemotherapy agents. These poorly-tolerated drugs could either be singly- or co-loaded onto the P1/P2 platform due to its synthetic versatility, and replace the cocktails used in approaches like this one to achieve comparable patient response while minimizing the co-morbidities that develop. Our ongoing studies are measuring the synthetic feasibility and preclinical efficacy of delivering other FDA-approved molecules using the P1/P2 platform.

## Conclusions

The results presented here clearly highlight the merit in using NAcGal-targeted G5 dendrimers to deliver DOX into hepatic cancer tissue. The cell-specific targeting combined with enzyme-mediated drug release confers improved therapeutic efficacy against tumors in mice over free DOX. At the same time, P1/P2 particles minimize DOX cardiotoxicity by maintaining complete cardiac function in treated mice. These findings suggest that P1 and P2 particles are promising nanoparticle formulations for improving the therapeutic index of DOX for advanced HCC patients in the clinic.

## Supporting information

S1 File General experimental procedures. (DOCX)

S1 Fig Compound 11 ^1^H NMR in D_2_O, 700 MHz. (DOCX)

S2 Fig Compound 11 MALDI spectrum. (DOCX)

S3 Fig Compound 12 ^1^H NMR in CD_3_SOCD_3_ + 3 drops of D_2_O; 700 MHz. (DOCX)

S4 Fig Compound 12 MALDI spectrum. (DOCX)

S5 Fig Compound 13 ^1^H NMR in CDCl_3_+ 3 drops of D_2_O; 700 MHz. (DOCX)

S6 Fig Compound 13 MALDI spectrum. (DOCX)

S7 Fig Particle size measurements. Individual replicates of particle size measurements as measured by dynamic light scattering (DLS).
(DOCX)

S8 Fig TEM images of P1 and P2 particles. TEM images of P1 (A, B) and P2 (C, D) particles at 50,000X (A,C) or 100,000X (B,D) zoom. The uranyl acetate-stained samples show isolated P1 and P2 particles with spherical morphology and diameters of 6.75 ± 0.23 nm and 7.12 ± 0.38 nm, respectively.
(DOCX)

S9 Fig Representative images of treated mice at day 21. (DOCX)

S10 Fig Survival curve of mice during cardiotoxicity experiment. (DOCX)
