# Supplementary material for: Dendrimer-doxorubicin conjugates exhibit improved anticancer activity and reduce doxorubicin-induced cardiotoxicity in a murine hepatocellular carcinoma model
Source: PLoS One. 2017 Aug 22;12(8):e0181944. doi: 10.1371/journal.pone.0181944 (PMC5567696; doi:10.1371/journal.pone.0181944)
Supplement: S1 File — (DOCX) [file pone.0181944.s001.docx]

**S1 Supporing Information**

**General Experimental Procedures**

**1. Synthesis of NAcGal-*c*PEG-G5-L(x)-DOX Particles:**

All reactions were carried out under nitrogen with anhydrous solvents in flame-dried glassware, unless otherwise noted. All glycosylation reactions were performed in the presence of molecular sieves, which were flame-dried right before the reaction under high vacuum. Solvents were dried using a solvent purification system and used directly without further drying. Chemicals used were reagent grade as supplied except where noted. Analytical thin-layer chromatography was performed using silica gel 60 F254 glass plates. Compound spots were visualized by UV light (254 nm) and by staining with a yellow solution containing Ce(NH_4_)_2_(NO_3_)_6_ (0.5 g) and (NH_4_)_6_Mo_7_O_24_·4H_2_O (24.0 g) in 6% H_2_SO_4_ (500 mL). Flash column chromatography was performed on silica gel 60 (230–400Mesh). NMR spectra were referenced using Me_4_Si (0 ppm), residual CHCl_3_ (δ ^1^H-NMR 7.26 ppm, ^13^C-NMR 77.0 ppm, CD_3_SOCD_3_ (δ ^1^H-NMR 2.49 ppm, ^13^C-NMR 39.5 ppm and D_2_O (δ ^1^H-NMR 4.65 ppm). Peak and coupling constant assignments are based on ^1^H-NMR.

**Characterization of anomeric stereochemistry:** The stereochemistry of the newly formed glycosidic linkages in N-acetyl galactosamine derivative was determined by J_H1_,_H2_ through ^1^H-NMR. Smaller coupling constants of J_H1_,_H2_ (below 4 Hz) indicate α linkages and larger coupling constants J_H1_,_H2_ (6.0 Hz or larger) indicate β linkages.

**Mass spectrometry (MS) analysis:** ESI-MS measurements were performed according to the published protocols on a Q-TOF Ultima API LC-MS instrument with Waters 2795 Separation Module (Waters Corporation, Milford, MA). All samples passed through an EagleEye HPLC C_18_ column, 3 mm × 150 mm, 5 μm at a flow rate of 0.5 mL/min with a linear gradient from 10% eluent B to 26% eluent B over eight minutes with the column temperature maintained at 45 °C. All injections were performed in the full-loop injection mode using a 10 μL sample loop. Eluent A consisted of a pure aqueous solution and eluent B contained 75% acetonitrile/25% aqueous solution (v/v). The following instrument settings were common for analyses S16 performed in both positive and negative ion modes: source temperature 120 °C, desolvation temperature 400 °C, collision energy 10 eV. When operated in negative ion mode, the mass spectrometer used the following instrument settings: capillary voltage 2.0 kV, cone voltage 35 V, extraction cone 4 V. The following instrumental parameters were used for data acquisition in positive ion mode: capillary voltage 3.5 kV, cone voltage 35 V. Sample concentrations were 1mg/mL. MALDI mass spectra were recorded on a Shimadzu Axima-CFR plus MALDI-TOF. The matrix used was 2,5-dihydroxy-benzoic acid (DHB) and Melittin from honeybee venom (M2272 from Sigma-Aldrich) as the calibration compound.

**We have reported the synthesis and analytical data for L3-DOX, L4-DOX linkers and compounds 1-10 in our previous work**^25^**. Below, we describe the synthesis and analytical data for compound 11, P1 & P2.**

*1.1 (N-((2R,3R,4R,5R,6R)-2-(2-(2-(2-aminoethoxy)ethoxy)ethoxy)-4,5-dihydroxy-6-(hydroxymethyl)tetrahydro-2H-pyran-3-yl)acetamide-PEG-NH-Cis-Ac)_16.2_-G5-(alkyne)_15_* (**11**):

Compound **9** (202 mg, 8.05 x 10^-2^ mmol, 18 eq) was dissolved in 12 mL of 0.1 M potassium phosphate buffer (pH 6.0) followed by addition of EDC.HCl (61 mg, 3.18 x 10^-1^ mmol, 1:4 eq with acid), HOBt (10.8 mg, 8.05 x 10^-2^ mmol, 1:1 eq with acid) and the reaction mixture was stirred at room temperature for 30 minutes. G5-(alkyne)_15_-(NH_2_)_115_ dendrimer **10** (135 mg, 4.49 x 10^-3^ mmol, 1 eq) was dissolved in 7 mL of MeOH and added to the reaction mixture followed by pH adjustment to 8.0, by drop wise addition of 0.5 M Na_2_CO_3_ solution. The reaction mixture was stirred for 36 hours at room temperature before dialyzing (MWCO 10kDa) the reaction solution against deionized water for 36 hours followed by lyophilization to obtain compound **11** as a light orange fluffy solid (305 mg) in 90% yield.

^1^H NMR (500 MHz, D_2_O): δ 1.80-1.85 (m, 36H, CH_3_, NHAc), 2.12-2.38 (m, 316H, G5-H, along with other ethylene dioxide protons), 2.40-2.56 (m, 120H, G5-H, un-overlapped G5 protons), 2.56-2.78 (m, 285H G5-H, along with other ethylene dioxide protons), 2.77 (bs, 9H, -OH), 2.83 (bs, 9H, -OH), 2.87 (bs, 27H), 2.94-3.00 (m, 42H), 3.00-3.24 (m, 364H, G5-H, along with other ethylene dioxide protons), 3.26-3.38 (m, 62H), 3.39-3.74 (m, 2782H, PEG-protons); 3.78 (bs 13H), 3.90 (bs 14H), 3.92 (bs 16.4H), 4.20 (bs 12H), 4.36 (bs 14H), 5.42 (d, 12H, *J* = 4.4 Hz), 5.77 (d, 1H, *J* = 7.4 Hz, H_1_), 7.22 (bs, NH protons), 7.53 (bs, NH protons), 7.63 (bs, NH protons), 7.94 (bs, NH protons).

NMR analysis: We took un-overlapped G5-protons as standard G5-120 protons at 2.40-2.56 ppm, and we obtained 2782 PEG- protons at 3.40-3.72 ppm. Each 2KDa PEG unit contains approximately 172 protons, and then we were able to attach 16.17 *cis*-Ac-PEG-NAcGAL units on to the G5 surface.

MALDI analysis: The molecular weight of the compound **9** is 2508, and compound **10** is 30033. The molecular weight observed for (alkyne)_15_-G5-(cis-Ac-PEG-NAcGAL) is 70,861 which has 40,828 daltons more than its parent dendrimer. This is attributed to *cis*-Ac-PEG-NAcGAL units; each *cis*-Ac-PEG-NAcGAL contributes 2508.2 daltons. Therefore obtained *cis*-Ac-PEG-NAcGAL functionality is 16.27 units.

*1.2(N-((2R,3R,4R,5R,6R)-2-(2-(2-(2-aminoethoxy)ethoxy)ethoxy)-4,5-dihydroxy-6-(hydroxymethyl)tetrahydro-2H-pyran-3-yl)acetamide-PEG-NH-Cis-Ac)_16.2_-G5-(L3-Dox)_13.1_* (**P1**):

First Flask: Sodium ascorbate (9.5 mg, 4.79 x 10^-2^ mmol), bathophenonthroline sulfonated sodium salt (SBP, 26.2 mg, 4.43 x 10^-2^ mmol) and Cu(I) 5 mg, 2.62 x 10^-2^ mmol) was dissolved THF:H_2_O, 1:1= 15 mL) and bubbled the nitrogen for 10 min.

Second Flask: L3-Dox-azide (17.6 mg, 1.97 x 10^-2^ mmol) was dissolved in THF (8 mL) and (*N*-Ac-Gal)_16.2_-G5-(alkyne)_15_ (**11**, 100 mg, 1.41 x 10^-3^ mmol) in H_2_O (7 mL) and bubbled the nitrogen for 10 min. The catalyst flask was heated to 75 ^o^C for 3-4 min (during this time the solution becomes red in color), cool down to RT, and syringe out the catalyst solution while bubbling the nitrogen and added to L3-dox-azide flask carefully (drop wisely), flushed the nitrogen one more time and closed the flask and covered with aluminum foil and stirred for 48 h. Stirring should be slow and constant around 350 rpm. After 2 days, the reaction mixture was transferred into dialysis cassette (10KDa) and dialyzed for 2 days against DI water followed by lyphilization afforded **P1**, approximately (105 mL, 1 mg/mL, 105 mg, 89.2% yield).

^1^H NMR (700 MHz, CD_3_SOCD_3_ + 2 drops of D_2_O): δ 0.72-0.85 (m, 80H, aliphatic protons), 0.90-1.36 (m, 340H, G5-protons), 1.36-1.56 (m, 105 H, G5-protons), 1.78-1.90 (m, 155H, G5-protons), 1.92-2.02 (m, 65H, including NHAc protons), 2.05-2.40 (m, 240H, G5-H, along with other ethylene dioxide protons), 2.52-2.60 (m, 46H), 2.62-2.80 (m, 120H), 2.95-3.20 (m, 210H), 3.20-4.40 (m, G5-protons, PEG-protons merged with DHO peak), 4.56 (s, 16H), 4.70 (s, 6H), 4.82 (s, 6H), 4.94 (s, 6H), 5.00 (s, 12H), 5.20 (s, 8H), 5.30 (s, 6H), 5.46 (s, 10H), 5.78 (s, 16H), 5.82 (s, 4H), 6.30 (s, 3H), 6.38 (s, 2H), 6.54 (s, 24H), 6.64 (s, 6H), 6.80 (s, 8H), 6.96 (s, 6H), 7.20 (s, 3H), 7.40 (s, 8H), 7.60-7.82 (m, 24H), 7.82-8.00 (m,10H), 13.24 (s, 2H, Dox-protons), 14.06 (s, 2H, Dox-protons).

MALDI analysis: The molecular weight of parent particle (alkyne)_15_-G5-(*cis*-Ac-PEG-NAcGAL)_16.2_ is 70,861. The molecular weight observed for (alkyne)_15_-(*cis*-Ac-PEG-NAcGAL)_16.2_-G5-L3-DOX is 82,577 which has 11,716 daltons more than its parent dendrimer. This is attributed to L3-DOX units; each L3-DOX contributes 893.2 daltons. Therefore obtained L3-DOX functionality is 13.1 units.

*1.5 (N-((2R,3R,4R,5R,6R)-2-(2-(2-(2-aminoethoxy)ethoxy)ethoxy)-4,5-dihydroxy-6-(hydroxymethyl)tetrahydro-2H-pyran-3-yl)acetamide-PEG-NH-Cis-Ac)_16.2_-G5-(L4-Dox)_13.4_* (**P2**):

First Flask: Sodium ascorbate (10.5 mg, 5.3 x 10^-2^ mmol), bathophenonthroline sulfonated sodium salt (SBP, 28.8 mg, 4.87 x 10^-2^ mmol) and Cu(I) 5.2 mg, 2.73 x 10^-2^  mmol) was dissolved THF:H_2_O, 1:1= 15 mL) and bubbled the nitrogen for 10 min.

Second Flask: L4-Dox-azide (20 mg, 2.17 x 10^-2^ mmol) was dissolved in THF (9 mL) and (*N*-Ac-Gal)_16.2_-G5-(alkyne)_15_ (**11**, 110 mg, 1.55 x 10^-3^ mmol) in H_2_O (8 mL) and bubbled the nitrogen for 10 min. The catalyst flask was heated to 75 ^o^C for 3-4 min (during this time the solution becomes red in color), cool down to RT, and syringe out the catalyst solution while bubbling the nitrogen and added to L4-dox-azide flask carefully (drop wisely), flushed the nitrogen one more time and closed the flask and covered with aluminum foil and stirred for 48 h. Stirring should be slow and constant around 350 rpm. After 2 days, the reaction mixture was transferred into dialysis cassette (10KDa) and dialyzed for 2 days against DI water followed by lyphilization afforded **P2**, approximately (115 mL, 1.0 mg/mL, 115 mg) in 88% yield.

^1^H NMR (700 MHz, CDCl_3_ + 3 drops of D_2_O): δ -1.50-0.4 (m, 320H, G5-H & aliphatic protons), 0.78-0.98 (m, 80H), 1.00-1.40 (m, 240 H, G5-protons), 1.40-2.10 (m, 440H, G5-protons), 2.05-2.15 (m, 24H, extended arm ethylene dioxide protons), 2.32-2.42 (m, 12H), 2.40 (m, 6H), 2.95-3.10 (m, 110H), 3.20-4.40 (m, G5-protons, PEG-protons merged with DHO peak), 4.56 (s, 16H), 4.78 (s, 6H), 5.00-5.18 (m, 24H), 5.20 (bs, 6H), 5.30-5.40 (m, 24H), 5.56 (s, 8H), 6.24 (s, 4H), 6.30 (s, 2H), 6.78 (s, 8H), 7.02 (s, 6H), 7.40 (s, 10H), 7.78 (s, 10H), 7.82 (s, 6H), 8.06 (s, 6H), 13.26 (s, 2H, Dox-protons), 14.02 (s, 2H, Dox-protons).

MALDI analysis: The molecular weight of parent particle (alkyne)_15_-G5--(*cis*-Ac-PEG-NAcGAL)_16.2_ is 710861. The molecular weight observed for (alkyne)_15_-(*cis*-Ac-PEG-NAcGAL)_16.2_-G5-L4-DOX is 83,277 which has 12,416 daltons more than its parent dendrimer. This is attributed to L4-DOX units; each L4-DOX contributes 923.2 daltons. Therefore obtained L4-DOX functionality is 13.4 units.

References:

- 1. (a) G. Tiruchinapally, Scott H. Medina, Maxim V. Chevliakov, Yasemin Y. Durmaz, Rachell N. Stender, William D. Ensminger, Donna S. Shewach, and Mohamed E.H. ElSayed, "Targeting hepatic cancer cells with PEGylated dendrimers displaying N-acetylgalactosamine and SP94 peptide ligands", Advanced Healthcare Materials, (**2013**) 2, 1337-1350. (b) S. H. Medina, Maxim V. Chevliakov, Gopinath Tiruchinapally, Yasemin Y. Durmaz, Sibu Kuruvilla, and Mohamed E.H. ElSayed, "Enzyme-activated nanoconjugates for tunable release of chemotherapeutic agents in hepatic cancer cells", Biomaterials, (**2013**) 34, 4655-4666.
