# Supplementary figures and images for: Dendrimer-doxorubicin conjugates exhibit improved anticancer activity and reduce doxorubicin-induced cardiotoxicity in a murine hepatocellular carcinoma model
Source: PLoS One. 2017 Aug 22;12(8):e0181944. doi: 10.1371/journal.pone.0181944 (PMC5567696; doi:10.1371/journal.pone.0181944)

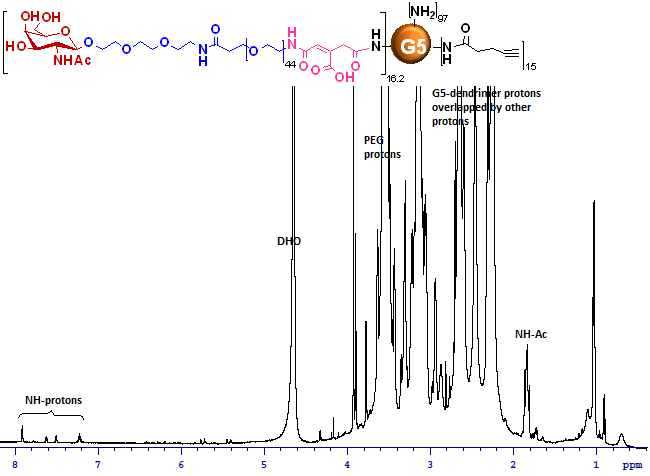


**S2 Fig. Compound 11 1H NMR in D2O, 700 MHz.**

Supplement: S1 Fig — (DOCX) [file pone.0181944.s002.docx]

**
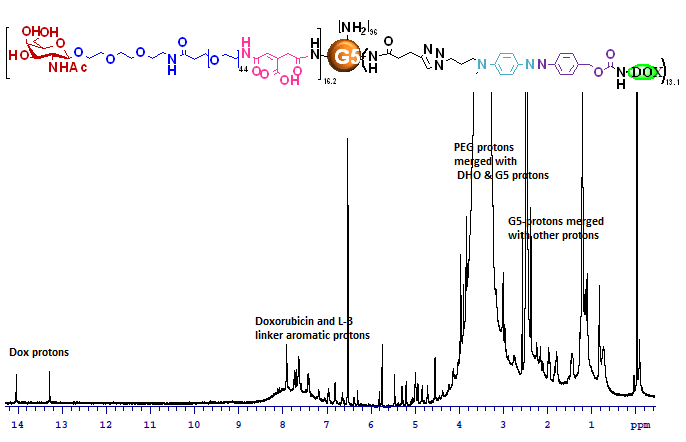
**

**S4 Fig. Compound 12 1H NMR in CD3SOCD3 + 3 drops of D2O; 700 MHz.**

Supplement: S3 Fig — (DOCX) [file pone.0181944.s004.docx]

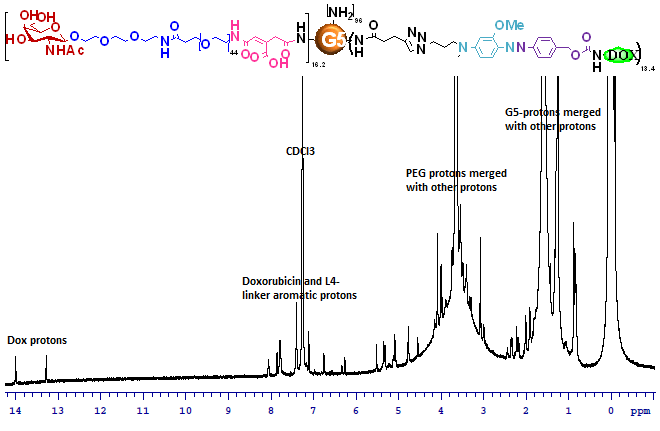


**S6 Fig. Compound 13 1H NMR in CDCl3+ 3 drops of D2O; 700 MHz.**

Supplement: S5 Fig — (DOCX) [file pone.0181944.s006.docx]

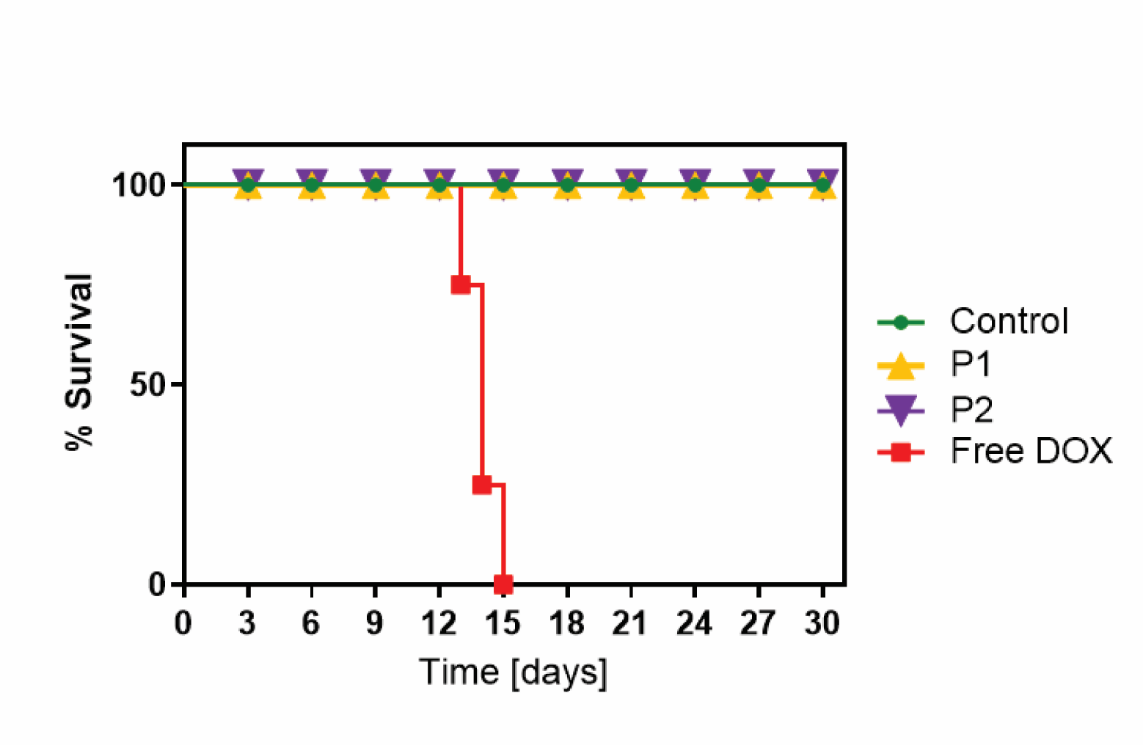


**S11 Fig. Survival curve of mice during cardiotoxicity experiment.**

Supplement: S10 Fig — (DOCX) [file pone.0181944.s011.docx]
