# Supplementary material for: Dendrimer-doxorubicin conjugates exhibit improved anticancer activity and reduce doxorubicin-induced cardiotoxicity in a murine hepatocellular carcinoma model
Source: PLoS One. 2017 Aug 22;12(8):e0181944. doi: 10.1371/journal.pone.0181944 (PMC5567696; doi:10.1371/journal.pone.0181944)
Supplement: S2 Fig — (DOCX) [file pone.0181944.s003.docx]

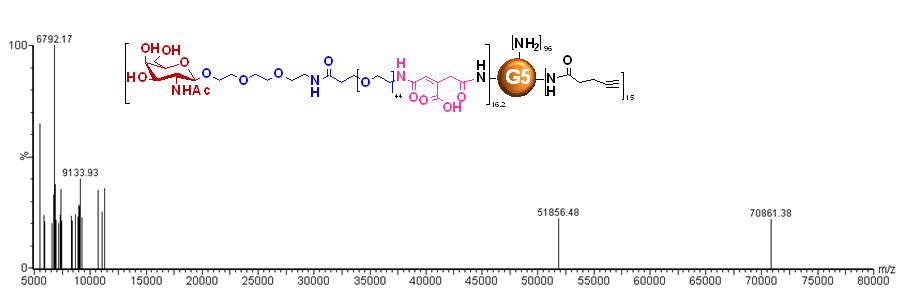


**S3 Fig. Compound 11 MALDI spectrum.**

Analysis:

1. The molecular weight of parent particle G5-(alkyne)_15_ is 30,033.
2. The molecular weight observed for _m_(NAcGal_β_-PEG*c*)-G5-(alkyne)_15_ is 70,861 which has 40,828 daltons more than its parent dendrimer. This is attributed to NAcGal-PEG*c* units; each NAcGal-PEG*c* contributes 2508.2 daltons. Therefore the obtained NAcGal-PEG*c* functionality is 16.2 units.
