# Supplementary material for: Dendrimer-doxorubicin conjugates exhibit improved anticancer activity and reduce doxorubicin-induced cardiotoxicity in a murine hepatocellular carcinoma model
Source: PLoS One. 2017 Aug 22;12(8):e0181944. doi: 10.1371/journal.pone.0181944 (PMC5567696; doi:10.1371/journal.pone.0181944)
Supplement: S4 Fig — (DOCX) [file pone.0181944.s005.docx]

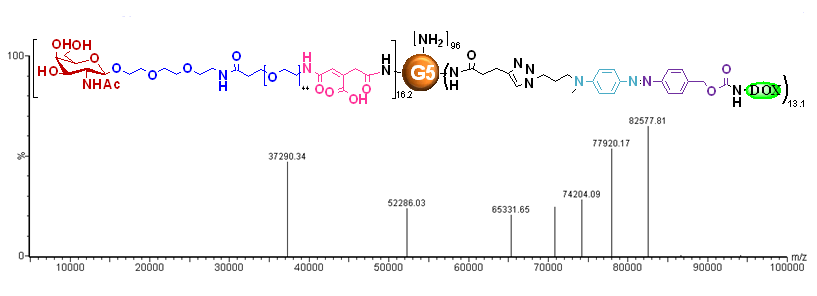


**S5 Fig. Compound 12 MALDI spectrum.**

Analysis:

1. The molecular weight of parent particle _16.2_(NAcGal_β_-PEG*c*)-G5-(alkyne)_15_ is 70,861.
2. The molecular weight observed for _16.2_(NAcGal_β_-PEG*c*)-G5-L3-DOX is 82,577 which has 11,716 daltons more than its parent dendrimer. This is attributed to L3-DOX units; each L3-DOX contributes 893.2 daltons. Therefore the obtained L3-DOX functionality is 13.1 units.
