# Supplementary material for: Dendrimer-doxorubicin conjugates exhibit improved anticancer activity and reduce doxorubicin-induced cardiotoxicity in a murine hepatocellular carcinoma model
Source: PLoS One. 2017 Aug 22;12(8):e0181944. doi: 10.1371/journal.pone.0181944 (PMC5567696; doi:10.1371/journal.pone.0181944)
Supplement: S6 Fig — (DOCX) [file pone.0181944.s007.docx]

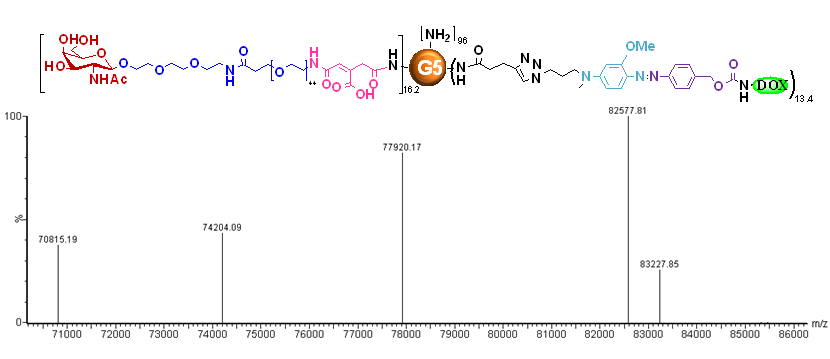


**S7 Fig. Compound 13 MALDI spectrum.**

Analysis:

1. The molecular weight of parent particle _16.2_(NAcGal_β_-PEG*c*)-G5-(alkyne)_15_ is 70,861.
2. The molecular weight observed for _16.2_(NAcGal_β_-PEG*c*)-G5-L4-DOX is 83,277 which has 12,416 daltons more than its parent dendrimer. This is attributed to L4-DOX units; each L4-DOX contributes 923.2 daltons. Therefore the obtained L4-DOX functionality is 13.4 units
