# Supplementary material for: Dendrimer-doxorubicin conjugates exhibit improved anticancer activity and reduce doxorubicin-induced cardiotoxicity in a murine hepatocellular carcinoma model
Source: PLoS One. 2017 Aug 22;12(8):e0181944. doi: 10.1371/journal.pone.0181944 (PMC5567696; doi:10.1371/journal.pone.0181944)
Supplement: S8 Fig — TEM images of P1 (A, B) and P2 (C, D) particles at 50,000X (A,C) or 100,000X (B,D) zoom. The uranyl acetate-stained samples show isolated P1 and P2 particles with spherical morphology and diameters of 6.75 ± 0.23 nm and 7.12 ± 0.38 nm, respectively. (DOCX) [file pone.0181944.s009.docx]

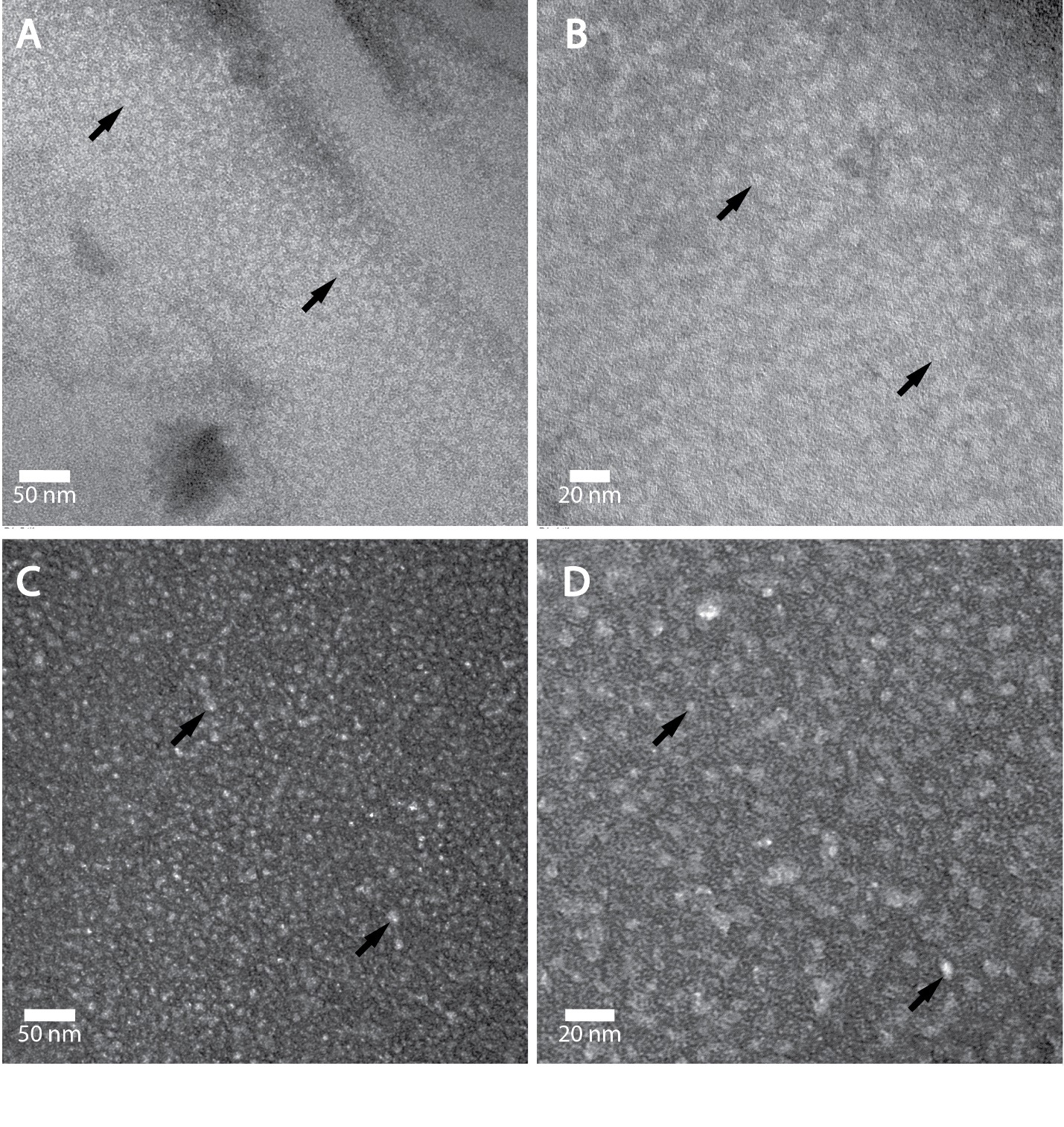


**S9 Fig. TEM images of P1 and P2 particles.** TEM images of P1 (A, B) and P2 (C, D) particles at 50,000X (A,C) or 100,000X (B,D) zoom. The uranyl acetate-stained samples show isolated P1 and P2 particles with spherical morphology and diameters of 6.75 ± 0.23 nm and 7.12 ± 0.38 nm, respectively.
