# Supplementary material for: Dendrimer-doxorubicin conjugates exhibit improved anticancer activity and reduce doxorubicin-induced cardiotoxicity in a murine hepatocellular carcinoma model
Source: PLoS One. 2017 Aug 22;12(8):e0181944. doi: 10.1371/journal.pone.0181944 (PMC5567696; doi:10.1371/journal.pone.0181944)
Supplement: S9 Fig — (DOCX) [file pone.0181944.s010.docx]

**S10 Fig. Representative images of treated mice at day 21.**
